# Bimetallic Silver–Gold Nanoplates for Photothermal Antimicrobial Therapy: Integrating High Efficiency with Stable and Multifunctional Design

**DOI:** 10.1021/acsanm.6c00304

**Published:** 2026-04-13

**Authors:** Javier Fernández-Lodeiro, Sebastian Tanco, Fernando Novio, Carlos Lodeiro, Julia Lorenzo

**Affiliations:** † BIOSCOPE Research Group, LAQV-REQUIMTE, Chemistry Department, NOVA School of Science and Technology (FCT NOVA), 119482 Universidade NOVA de Lisboa, Caparica 2829-516, Portugal; ‡ PROTEOMASS Scientific Society, Costa de Caparica 2825-466, Portugal; § Institut de Biotecnologia i de Biomedicina, Departament de Bioquímica i Biologia Molecular, 16719 Universitat Autònoma de Barcelona, Cerdanyola Del Vallès, Barcelona 08193, Spain; ∥ CIBER de Bioingeniería, Biomateriales y Nanomedicina, Instituto de Salud Carlos III, Campus UAB, Bellaterra 08913, Spain; ⊥ Chemistry Department, Faculty of Sciences, Universitat Autonòma de Barcelona, Cerdanyola Del Vallès, Barcelona 08193, Spain

**Keywords:** Ag–Au nanoplates, plasmonic nanomaterials, silica coating, photothermal, antimicrobial

## Abstract

Antimicrobial photothermal therapy requires nanomaterials that combine high plasmonic performance, colloidal stability, and scalable synthesis. In this work, we prepared bimetallic silver nanoplates partially coated with an ultrathin gold layer in a larger-volume batch (160 mL), while preserving stable optical properties and tunable photothermal performance in the biological near-infrared range. The resulting silver–gold nanoplates efficiently convert near-infrared light into heat and exert potent antimicrobial effects against *Pseudomonas aeruginosa* and *Staphylococcus aureus*. Systematic evaluation showed that thiol-terminated polyethylene glycol functionalization preserves the optical response of the nanoplates while improving colloidal stability in biologically relevant media. Furthermore, we investigated the trade-off between multifunctionality, stability, and photothermal efficiency observed after silica coating followed by covalent BSA conjugation, which increases surface functionalization versatility but reduces antimicrobial performance under the tested conditions. This work presents an efficient route to synthesize bimetallic Ag–Au nanoplates in large-volume batch for noninvasive antimicrobial photothermal applications and outlines key design principles to guide the development of next-generation multifunctional plasmonic nanoplatforms for antimicrobial therapy.

## Graphical abstract

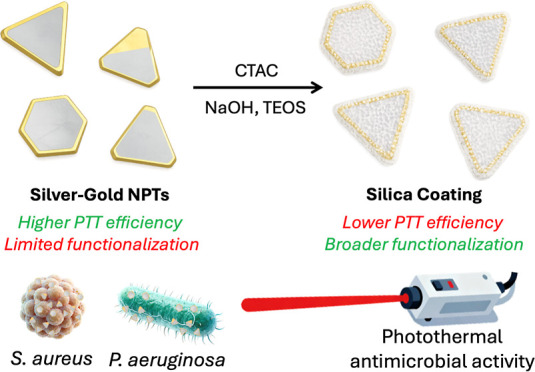

## 1 Introduction

Efficient, noninvasive treatment of bacterial infections, especially those involving multidrug-resistant strains and resilient biofilms, fundamentally relies on the innovative design of nanomaterials.^1^ Recent advances in nanotechnology have positioned plasmonic nanoparticles as powerful and versatile photothermal agents, capable of converting light into highly localized heat and thereby opening new possibilities for more effective antibacterial therapies.^2–4^ Upon irradiation, these nanoparticles increase bacterial membrane permeability and can promote oxidative stress via reactive oxygen species generation, culminating in irreversible cell damage and robust pathogen eradication.^3–5^

The development of precisely engineered anisotropic plasmonic nanostructures, including nanorods,^6^ nanoplates (NPTs),^7^ nanocages,^8^ nanostars,^9^ among others, has further expanded the frontier of photothermal therapy (PTT) and targeted antibacterial strategies. By tailoring their optical properties, these materials efficiently absorb light within the near-infrared (NIR) window (700–1000 nm), enabling deep tissue penetration and spatially controlled heating with minimal invasiveness. This capability is central to the advancement of photothermal platforms designed for selective destruction of pathogenic bacteria and malignant cells.^10–12^

While anisotropic gold nanostructures, such as nanorods,^6^ nanostars^13^ or NPTs,^14^ are well established for their outstanding photothermal conversion and broad use in biomedical research, recent literature increasingly highlights the promise of silver-based anisotropic nanoparticles (NPs).^15,16^ Anisotropic silver NPs, most notably NPTs, exhibit distinctive physicochemical properties due to their shape, including enhanced surface area and highly tailored localized surface plasmon resonance (LSPR).^7,17^ These attributes contribute to improved photothermal conversion and foster strong interactions with biological membranes, supporting both direct bactericidal action and efficient heat delivery upon irradiation in antimicrobial applications.^18–21^ Recent studies have further highlighted the structure-dependent plasmonic behavior and biomedical potential of anisotropic Ag- and Au-based nanostructures for photothermal applications.^22^

Despite such progress, the application of silver NPTs for biomedical PTT continues to present challenges. Their high surface reactivity and sensitivity to oxidation often demand additional strategies to increase stabilization, as aggregation or oxidation can shift optical properties and reduce photothermal therapeutic efficacy.^18^ Moreover, postsynthetic functionalization remains complex, particularly when seeking biomolecular conjugation for targeted therapies or improved colloidal stability in biologically relevant media. These limitations have prompted the use of stabilizing coatings, such as silica^23–25^ or a thin gold shell,^26^ that provide both chemical protection and modularity for functionalization, while supporting colloidal stability.

In summary, although silver NPTs show great promise for photothermal biomedical applications, significant challenges remain in their development. Advancing robust and reproducible synthetic methods that reliably produce silver-based NPTs with enhanced stability and functional versatility is critical. Addressing these limitations will be essential to unlocking their full potential as effective and tunable agents for photothermal antibacterial treatments.

In this work, we describe the synthesis of silver NPTs and their subsequent coating with a partial thin gold layer, a strategy that improves their physicochemical stability while enabling the larger-volume preparation of the bimetallic nanoplate platform. In parallel, we prepared a second set of NPTs coated with silica to evaluate how different surface modifications influence their antimicrobial efficacy. We then systematically compared the photothermal antibacterial activity of these NPTs against clinically relevant pathogens, including *Pseudomonas aeruginosa* and *Staphylococcus aureus*. The overall synthetic strategy and the two surface-functionalization routes investigated in this work are summarized in Scheme 1. Together, our results provide insights into how the nature of surface coatings influences colloidal stability, photothermal behavior and biological efficacy, providing practical guidelines for the design of next-generation plasmonic NPTs for antimicrobial photothermal therapy.

**Scheme 1:**
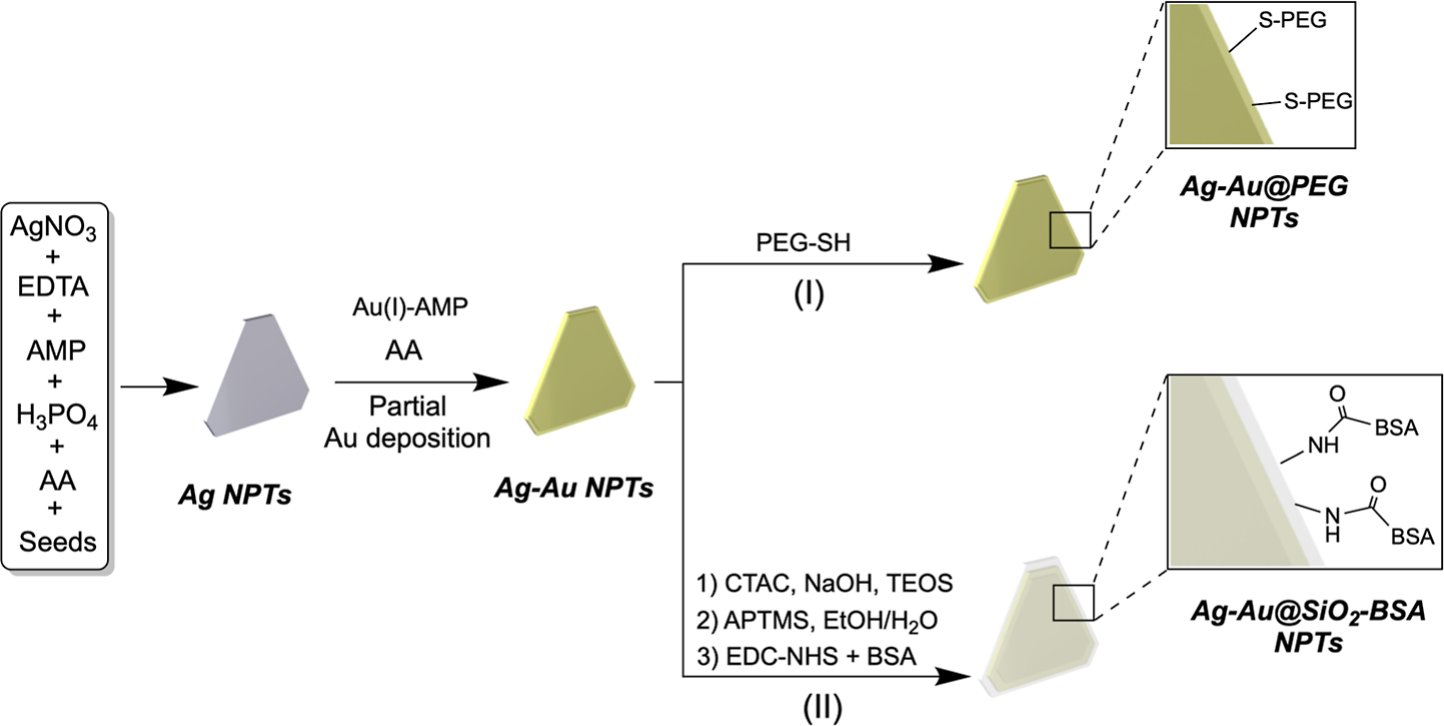
Schematic Representation of the Synthesis and Surface Functionalization of Bimetallic Ag–Au Nanoplates^a^

a Ag NPTs were synthesized and converted into Ag–Au NPTs through Au­(I)-AMP-mediated partial Au deposition. Route (I) corresponds to PEG-SH functionalization to yield Ag–Au@PEG NPTs, whereas route (II) corresponds to silica coating followed by APTMS functionalization and BSA conjugation to yield Ag–Au@SiO_2_–BSA NPTs.

## 2 Experimental Section

### 2.1 Materials

Silver nitrate (AgNO_3_, ≥99%, CAS: 7761-88-8), tetrachloroauric acid trihydrate (HAuCl_4_·3H_2_O, ≥99%, CAS: 16961-25-4) were obtained from Alfa Aesar. Ethylenediaminetetraacetic acid tetrasodium salt hydrate (EDTA, ≥99%, CAS: 194491-31-1), adenosine 5′-monophosphate disodium salt (AMP, ≥99%, CAS: 4578-31-8), phosphoric acid (H_3_PO_4_, ≥99%, CAS: 7664-38-2), l-ascorbic acid (AA, ≥99%, CAS: 50-81-7), sodium hydroxide (NaOH, ≥98%, CAS: 1310-73-2), sodium borohydride (NaBH_4_, ≥99%, reagent plus, CAS: 16940-66-2) were obtained from Merck. Tetraethyl orthosilicate (TEOS, 99.99% metal basis, CAS: 78-10-4), aminopropyltrimethoxysilane (APTMS, 98%, CAS: 919-30-2), *N*-hydroxysuccinimide (NHS, 98%, CAS: 6066-82-6) and 1-ethyl-3-(3-(dimethylamino)­propyl)­carbodiimide hydrochloride (EDC, 99%, CAS: 25952-53-8) were obtained from Thermo Scientific. Ultrapure water Type I, produced using a Millipore Simplicity UV. Yeast extract and Tryptone were obtained from Condalab. Agar BIOS special LL, bacteriologic was obtained from Biolife. PBS tablets for PBS buffer preparation were obtained from Medicago. Sterile Ø90 mm polystyrene Petri dishes were obtained from Labbox.

### 2.2 Characterizations

UV–visible–NIR extinction spectra were recorded using a JASCO 770 spectrophotometer or a Cary 60 UV–vis spectrophotometer using quartz cells of 1 cm optical path length. Low-resolution TEM images were obtained with a JEOL JEM 1010 TEM operating at an acceleration voltage of 100 kV, utilizing copper or gold grids with thin carbon film to deposit the samples (CACTI-UVigo User Facilities) or using a JEOL 2011 TEM (200 kV) (Servei de Microscòpia i Difracció de Raigs X, UAB).

Scanning transmission electron microscopy (STEM) coupled with energy-dispersive X-ray spectroscopy (EDS) experiments was performed to study the chemical composition of 2 samples. High-angle annular dark field (HAADF) STEM images were acquired using a probe-corrected FEI Titan ChemiSTEM. The images, with a resolution of 2048 × 2048 pixels, were recorded at a convergence angle of 21 mrad with a pixel dwell time of 4 μs. EDS mapping was performed using the same instrument with a Super-X EDS detector. Iterative maps of 1024 × 1024 pixels were recorded with a dwell time of 5 μs per pixel at 200 keV, and with collection times of 15 min. The Esprit 1.9 software was used for acquiring and processing the STEM-EDS data.

Elemental determination was performed by ICP–MS (7900, Agilent). Samples were digested using a microwave digestion system (Ultrawave, Milestone) with a mixture of concentrated HCl and HNO_3_.

Pump system, model 100 NEW ERA, was used to carry out the synthesis of the seeds. The pH of the reactions was measured using a pH meter (HACH-SensION pH31).

For laser irradiation, we used an MDL-III-785 nm-300 mW diode laser emitting a square beam pattern characteristic of multimode diode emission. Laser power was measured at 785 nm using a LASERPOINT A-2-D12-HPB-U thermal sensor positioned at the same distance used in the PTT analysis, and the power density was calculated from the measured power and the laser spot area.

Electron micrograph images were analyzed using ImageJ software. Bacterial quantification results were processed and statistically analyzed with GraphPad Prism software.

### 2.3 Methods

#### 2.3.1 Synthesis of Silver Seeds

Silver seeds were synthesized at room temperature (21 °C). Briefly, 14.7 mL of water and 300 μL of freshly prepared 20 mM NaBH_4_ were placed in a 25 mL opaque vial under vigorous magnetic stirring. Then, 5 mL of a freshly prepared aqueous solution containing 200 μL of 20 mM EDTA and 100 μL of 20 mM AgNO_3_ was added dropwise at 1 mL/min. The mixture was stirred for 2 h, and the resulting seed solution was stored at ∼4 °C in the dark for 8 h to allow decomposition of excess NaBH_4_. The seeds were used without further purification and remained suitable for use for at least 1 week when stored at ∼4 °C.^7^

#### 2.3.2 Synthesis of Silver NPTs (320 mL)

Silver NPTs were synthesized in 320 mL based on a prior report^7^ using an appropriate round-bottom flask under magnetic stirring and ambient laboratory lighting at room temperature (21 °C). Stock solutions of AgNO_3_, AMP, EDTA, AA and H_3_PO_4_ at 20 mM concentration were used, maintaining a total reaction volume of 320 mL. Briefly, 2.4 mL of AgNO_3_, 9.6 mL of EDTA, 2.4 mL of AMP, and 1.44 mL of phosphoric acid were combined with 299.36 mL of water. After stirring for 1 min, 2.4 mL of ascorbic acid (AA) and 2.4 mL of silver seed solution were rapidly injected. The mixture was magnetically stirred at room temperature for 90 min.

#### 2.3.3 Synthesis of Silver@Gold Bimetallic NPTs

For the synthesis of bimetallic NPTs, 160 mL of the silver nanoplates colloidal solution were transferred to a round-bottom reaction flask. Four consecutive additions of 10 mL of a freshly prepared gold precursor solution were then performed. Each 10 mL aliquot contained 75 μL of HAuCl_4_, 300 μL of AMP, and 125 μL of AA, corresponding to an Au­(III)/AMP/AA molar ratio of 1:4:1.67. The precursor solution was added at 0.25 mL min^–1^ using a syringe pump under vigorous magnetic stirring. After completing the last addition, the reaction mixture was left under moderate stirring for 24 h. Subsequently, 4 mL of 20 mM AMP were added, and the suspension was stirred for an additional 1 h. The resulting bimetallic NPTs were purified by two consecutive centrifugation steps at 4500 rpm for 30 min, resuspending the pellets in 2 mM NaOH to obtain purified colloids. Centrifugation was carried out in 15 mL tubes, loading 10 mL of colloid per tube, and the final purified NPTs were resuspended in 80 mL of 2 mM NaOH.

#### 2.3.4 Functionalization with PEG-SH

Thirty mL of purified NPTs in 2 mM NaOH were mixed with 3 mL of PEG-SH (1 mg/mL) and stirred for 24 h. Subsequently, the NPTs were washed three times with water to remove excess, unbound polymer.

#### 2.3.5 Silica Coating of Silver@Gold NPTs

Silica coating was applied to 40 mL of purified Ag–Au NPTs (functionalized only with AMP on the surface) suspended in 2 mM NaOH, within a total reaction volume of 120 mL (excluding the ethanol volume containing the silica precursor). A round-bottom flask was charged with 77.68 mL of water and 1.92 mL of 50 mM CTAC under moderate magnetic stirring. Forty milliliters of the Ag–Au NPTs in 2 mM NaOH were added dropwise under stirring. Subsequently, 0.4 mL of 0.1 M NaOH was injected, and the solution was stirred moderately for 6 h. Finally, 4 mL of ethanol containing 60 μL of TEOS was added dropwise in 1 mL portions every 15 min under vigorous stirring. The reaction was allowed to proceed for 24 h with moderate stirring. The coated NPTs were washed through consecutive centrifugation cycles in ethanol and finally resuspended in 60 mL of ethanol for storage at 4 °C.

#### 2.3.6 Functionalization of Silica NPTs with Terminal Amines via Silane Condensation

Purified NPTs (40 mL in ethanol) were transferred to a round-bottom reaction flask. Then, 8 μL of APTMS was added under vigorous stirring, followed by 2 mL of water. The reaction mixture was stirred vigorously for 4 h. During the first 2 h, NPTs aggregation occurred, which was reversed by ultrasonic irradiation for 30 s to redisperse the particles. Stirring was then continued for the remaining 2 h. Once the reaction had finished, NPTs were washed multiple times with ethanol by centrifugation and finally resuspended in 42 mL of ethanol for storage at 4 °C.

#### 2.3.7 Functionalization of Silica-Coated Ag–Au NPTs with Bovine Serum Albumin via Carbodiimide Chemistry

Forty milliliters of Ag–Au@SiO_2_–NH_2_NPTs were washed twice with 50 mM MES buffer (pH 6) and resuspended in 30 mL MES buffer. Stock solutions of BSA (3.6 mg/mL), EDC (3.2 mg/mL), and NHS (4.7 mg/mL) were prepared in MES buffer (50 mM, pH 6). A round-bottom flask was charged with 14 mL of MES buffer, followed by the addition of 115 μL BSA, 435 μL EDC, and 125 μL NHS. The mixture was stirred for 30 min to activate carboxyl groups. Subsequently, 30 mL of NPTs in MES buffer were added dropwise under stirring. The reaction proceeded for 3 h, after which the pH was adjusted to 7 with aliquots of 0.5 M NaOH, maintaining stirring for an additional 1 h. Following the reaction, NPTs were washed with 10 mM MES buffer and finally resuspended in 30 mL MES buffer for storage at 4 °C. Prior to use in bacterial assays, the NPTs were washed twice with sterile PBS buffer (pH 7.4).

#### 2.3.8 Photothermal Analysis of NPTs

For photothermal measurements, 100 μL of nanoplate dispersions at different Ag concentrations (determined by ICP–MS), prepared either in water or PBS, were transferred into 1.5 mL Eppendorf tubes, tightly capped, and irradiated at power densities of 2.7 or 1.4 W/cm^2^. Temperature changes were monitored using a FLIR thermal camera. For the determination of photothermal conversion efficiency from heating–cooling experiments, NPT dispersions were placed in 1.5 mL Eppendorf tubes left uncapped to allow thermal equilibration with the ambient environment during the cycles.

### 2.4 Bacterial Studies

#### 2.4.1 Mid Logarithmic Phase Bacterial Culture Preparation

Single colonies of *S. aureus* or *P. aeruginosa* were inoculated into 10 mL of Luria–Bertani (LB) broth in sterile culture tubes and incubated at 37 °C with agitation (200 rpm) overnight (ON) in an orbital shaker to generate (ON) precultures. From each ON preculture, a 400 μL aliquot was transferred into 10 mL fresh LB medium and incubated at 37 °C and 200 rpm until the cell suspension reached an optical density (OD) at 600 nm (OD_600_) of 0.6, as measured with a UV–vis spectrophotometer. This value corresponds to approximately 10^9^ colony-forming units (CFU)/mL. The time required to achieve OD_600_ = 0.6 was ca. 70 min for *S. aureus* and ca. 130 min for *P. aeruginosa*.

#### 2.4.2 Preparation of Working Bacterial Suspensions in PBS

For each strain, 1 mL of the mid logarithmic phase culture was harvested by centrifugation in 1.5 mL microcentrifuge tubes. Centrifugation conditions were as follows: 14,800 rpm for *S. aureus* and 8000 rpm for *P. aeruginosa*, for 5 min at room temperature. The bacterial pellet was resuspended in 1 mL of sterile PBS, and this washing step was repeated twice more (total of three washes with PBS). The washed bacterial suspension was diluted 1/100 in PBS, yielding a concentration of *ca*. 10^7^ CFU/mL.

#### 2.4.3 Preparation of Test Solutions

A 10 μL aliquot of the diluted bacterial suspension (ca. 10^7^ CFU/mL) was mixed with 90 μL of NPTs at varying concentrations to achieve final silver concentrations of 7.45, 14.9, 29.8, or 44.7 ppm prepared in PBS. Stock concentrations of NPTs were determined by ICP–MS to establish the explored Ag levels in this work. Control samples were prepared by mixing 10 μL of bacterial suspension with 90 μL of PBS. All conditions were assessed in triplicate.

#### 2.4.4 Incubation and Irradiation

Both mixtures were stored in the dark for a total of 5 h. During this incubation period, selected samples were irradiated with a 785 nm laser at power densities of either 2.7 or 1.4 W/cm^2^ for 5, 10, 15, or 20 min, as described in the main text.

#### 2.4.5 Colony Counting

After irradiation, bacterial viability was assessed. Treated cultures were serially diluted in PBS and LB according to the following scheme.

(a) *S. aureus*: dilution series were 1/2 in PBS and 1/10 in LB.
(b) *P. aeruginosa*: dilution series were 1/10 in PBS and 1/10 in LB.

From the final dilution, 50 μL aliquots were plated on LB agar plates and incubated overnight at 37 °C. Colony-forming units were counted the following day.

#### 2.4.6 SEM Sample Preparation for Bacterial Imaging

For SEM analysis, *P. aeruginosa* suspensions were subjected to the same irradiation conditions used in the antibacterial experiments, either in the absence or presence of nanoplates. After irradiation, cells were recovered by centrifugation, resuspended in PBS supplemented with 4% formaldehyde, and fixed for 30 min at room temperature. The samples were then washed twice by centrifugation using water as the resuspension medium. The resulting bacterial suspensions were deposited onto silicon wafers, dried, and sputter-coated with a thin Pt layer prior to SEM imaging.

## 3 Results

Building on our previous work on stabilizing silver NPTs by gold deposition, in which we achieved the controlled synthesis of bimetallic silver–gold nanoplates (Ag–Au NPTs) with tailored morphology and composition,^27^ and discussed the AMP-mediated Au deposition process in detail, we now extend this approach to evaluate their photothermal performance. Specifically, the present work focuses on Ag–Au NPTs featuring a thin, incomplete gold coating, with particular emphasis on their potential antimicrobial applications.

We initiated this study by examining the synthesis of the Ag–Au NPTs in large-volume batch (8 times over our previous effort). Whereas our earlier work focused on 20 mL reaction batches,^27^ the present approach achieves an 8-fold increase in reaction volume, yielding 160 mL of well-defined bimetallic NPTs that exhibit highly consistent and predictable optical properties. Establishing reproducible and scalable synthetic methodologies is critical for translating nanoscale materials from proof-of-concept stages to technological applications in catalysis, photonics, and biomedicine, where large quantities of structurally uniform nanostructures are indispensable.

The synthesis parameters were optimized to produce NPTs with a mean size of approximately 100 nm, following a stoichiometric ratio of Ag/AMP/EDTA = 1:1:4. To generate a thin gold coating, we used 25% of Au­(I)-AMP as gold precursor (based on the silver content used to grow AgNPTs). The gold precursor, stabilized with AMP, was prereduced with ascorbic acid immediately before addition. As discussed in our previous study, this AMP-mediated Au­(I) precursor enables controlled Au deposition while limiting extensive galvanic replacement and preserving the nanoplate morphology.^27^

Figure 1A shows the optical response of the purified Ag–Au NPTs, exhibiting a dominant LSPR peak centered at ∼845 nm, well within the biological transparency window (700–1000 nm). The NPTs can be readily purified by centrifugation without any alteration to their optical properties, preserving their plasmonic response during storage at 4 °C for at least 6 months, as shown by the minimal shift in the LSPR maximum (∼5 nm) (Figure S1). Low-magnification transmission electron microscopy (TEM) images confirm that the NPTs retain their morphology and structural integrity after scaling, with a mean size of 102 ± 10.2 nm (Figure 1B,C) and HAADF and EDS demonstrate the bimetallic composition with low content of Au at the surface of NPTs (Figure 1D–F). Inductively coupled plasma mass spectrometry (ICP–MS) analysis of the bimetallic NPTs revealed an Au content of 5.4% relative to Ag. Together, these observations are consistent with the formation of Ag–Au NPTs bearing a low Au content associated with a thin, partial surface coating, in agreement with our previous structural analysis of the same AMP-mediated deposition strategy.^27^

**Figure 1:**
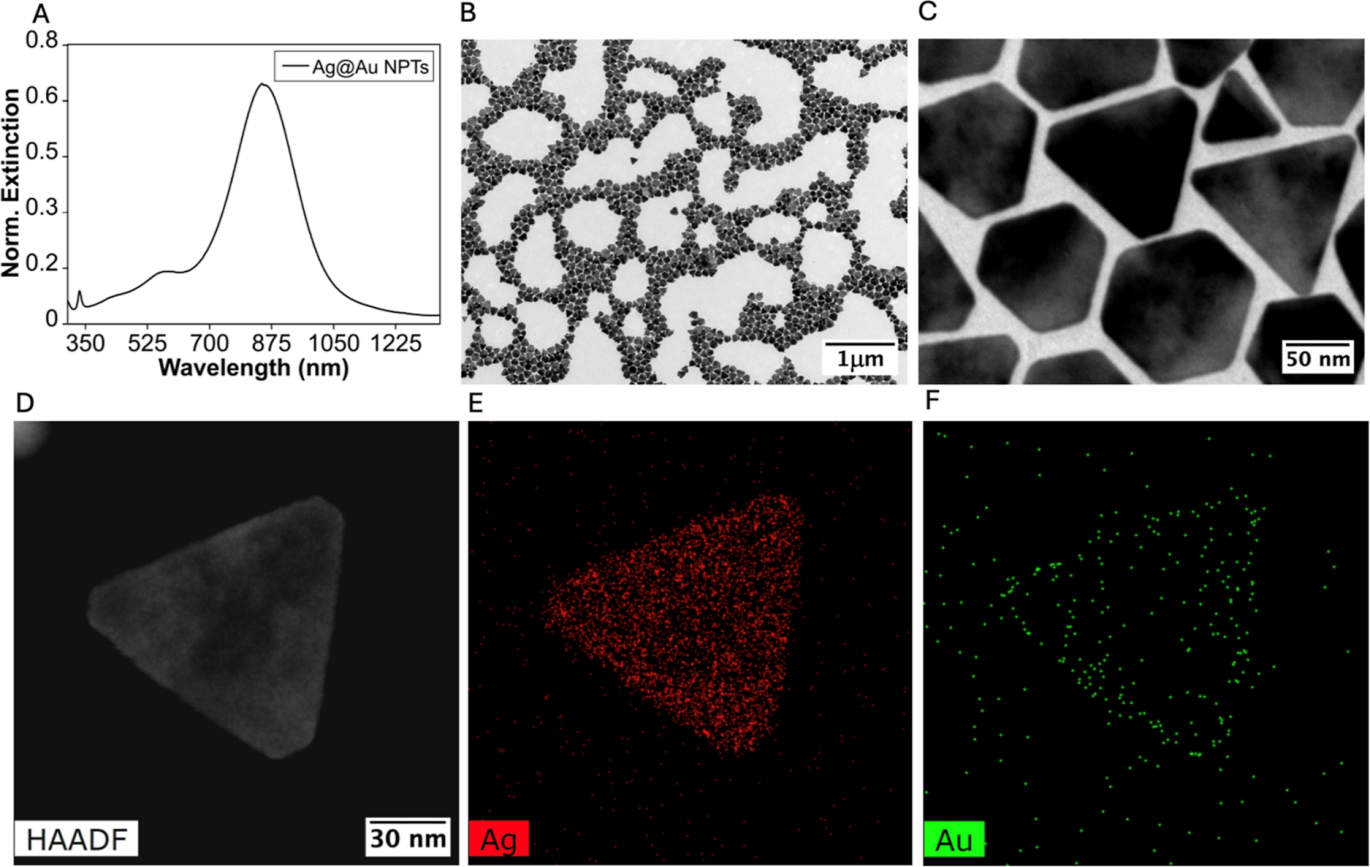
(A) Extinction spectra of purified Ag–Au NPTs. (B,C) TEM images at different magnifications of Ag–Au NPTs. (D–F) HAADF and EDS images of a single NPT.

Effective evaluation of photothermal activity under biological conditions requires stabilization of NPTs in physiological buffers, such as phosphate-buffered saline (PBS). NPTs stabilized solely with adenosine monophosphate (AMP) exhibit inadequate colloidal stability in PBS. The ionic strength of PBS screens the electrostatic repulsion conferred by AMP phosphate groups, resulting in aggregation and limiting their reliability for biological assays (Figure S2A). To address this challenge, thiol-terminated polyethylene glycol (PEG-SH), a widely used polymer with low cytotoxicity was employed to functionalize the NPTs.^28,29^ PEG-SH modification enables efficient redispersion and provides prolonged colloidal stability in PBS. Furthermore, it preserves the intrinsic optoelectronic properties of NPTs, creating a robust platform suitable for antimicrobial applications (Figure S2B,C).

To investigate the photothermal behavior of the synthesized bimetallic NPTs, their temperature response was evaluated in aqueous solution under 785 nm laser irradiation. Using this setup, photothermal effects were systematically analyzed at two distinct irradiation power densities and across varying NPT concentrations. The temperature rise in aqueous solution was measured using a FLIR thermal camera, a widely validated technique recognized for its high sensitivity and reproducibility in assessing nanomaterial heating dynamics under laser irradiation.^30,31^ As a control, pure water (lacking NPTs) was subjected to laser exposure at an equivalent power density to that employed in the antimicrobial assays. In these control experiments, the temperature increase remained within 1–2 °C after 20 min of continuous irradiation, indicating negligible photothermal effect in the absence of NPTs (Figure S3).

In our study, silver NPTs with a minimal gold coating were employed to maximize silver core stability while preserving robust plasmonic properties. At the highest tested concentration (59.6 ppm Ag, ICP–MS), laser irradiation at 2.7 W/cm^2^ resulted in the temperature increasing from room temperature to a steady-state temperature plateau of approximately 71 °C (Figure S4). This value exceeds the threshold required to induce most biologically significant photothermal effects. Temperature increases showed direct dependence on NPT concentration (Figure 2A), enabling precise thermal tuning. At low concentrations (i.e., 7.45 ppm Ag), only moderate heating was observed, with temperatures reaching approximately 43 °C. This controllable photothermal response is critical for biological and biomedical applications requiring precise thermal regulation, such as targeted drug delivery and PTT.^32^

**Figure 2:**
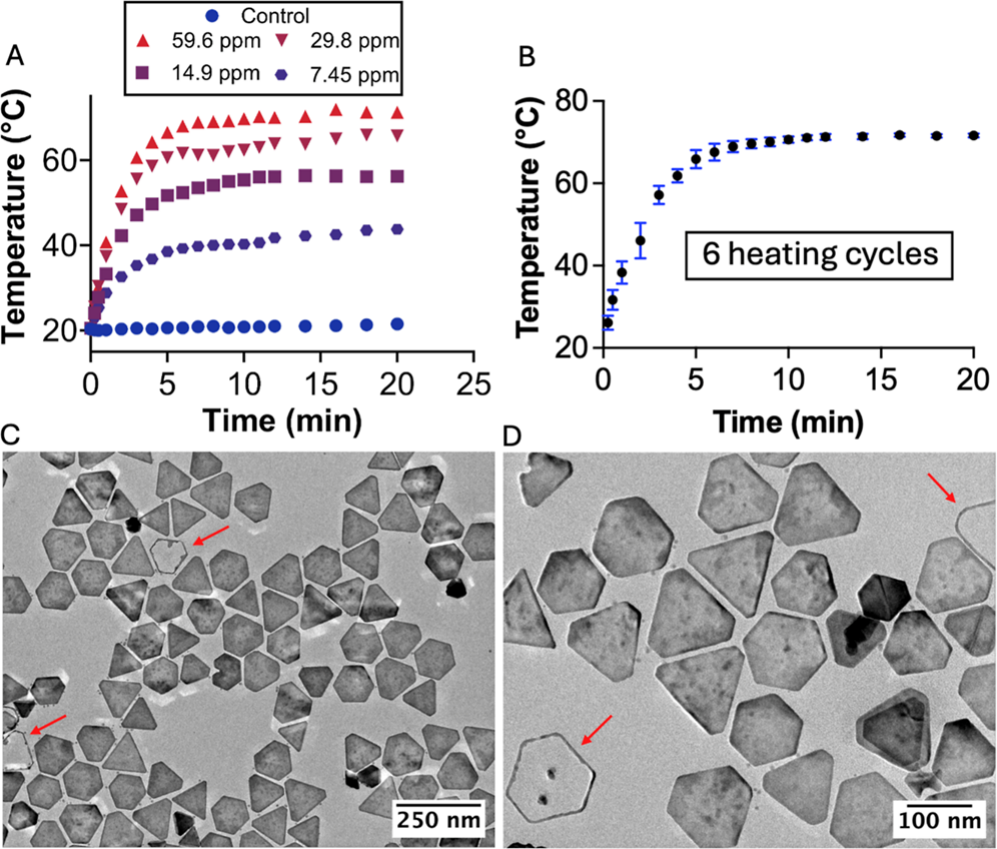
(A) Photothermal response of a representative replicate of NPTs in water at different concentrations, ranging from 7.45 to 59.6 ppm Ag (blue spots correspond to water control) (B) photothermal stability over six consecutive irradiation cycles at 59.6 ppm Ag concentration; error bars represent the standard deviation of the six PTT cycle measurements. (C,D) HRTEM images of NPTs subjected to six consecutive irradiation cycles at different magnifications. (red arrows indicate some of NPTs that suffered Ag-oxidation).

The impact of bimetallic structure and second metal coverage is supported by recent studies. Gargiulo et al. show that complete shell coatings of a secondary metal over a plasmonic core greatly dampen surface plasmon resonance and photothermal response, whereas satellite-like or localized coatings retain strong light absorption and heat generation.^33^ Borah and Verbruggen. demonstrate that bimetallic Au–Ag alloyed or core–shell particles exhibit different spectral and photothermal characteristics, with minimal shell preserving superior plasmonic and photothermal behavior compared to full coverage.^34^ Comparative experimental and theoretical studies also confirm that silver NPs provide more intense LSPR and enhanced photothermal efficiency over gold, especially with minimal noble metal doping or coating.^34,35^

Thus, by restricting gold coating to a thin and incomplete stabilizing layer, our bimetallic nanoplates maintain high LSPR intensity and should maximize photothermal conversion.

Notably, the thermal cycling experiments confirmed the exceptional stability of the bimetallic NPTs, as six sequential laser irradiation cycles produced reproducible temperature profiles (Figures 2B and S5A). TEM images and optical response of NPTs exposed to repeated photothermal treatments in aqueous media revealed minimal structural and optical changes, demonstrating high stability under irradiation (Figures 2C,D and S5B). Nonetheless, slight oxidation of the silver domains was observed, mainly along the basal facets, while the gold-enriched edges and corners remained intact, with a small fraction of NPTs undergoing transformation into Au-rich nanoring structures (Figure 2C,D).

The photothermal conversion efficiency of the bimetallic NPTs was determined using the Roper cooling-curve method, a widely accepted approach for quantifying light-to-heat conversion in plasmonic nanomaterials.^36^ Experimental temperature data were collected during the heating and subsequent cooling phases of the NPT solution with an optical density of 0.89 at 785 nm (Figure 3A). The postirradiation cooling curve was fitted with an exponential decay function to extract the system time constant (τ), which was subsequently used to calculate the product of the heat transfer coefficient and the illuminated surface area (*hA*). The photothermal conversion efficiency (η) was computed using the equation

$$\eta =\frac{hS\left(T_{\max}-T_{amb}\right)-Q_{dis}}{I\left(1-10^{-A_{\lambda }}\right)}$$

where *T*_max_ is the maximum temperature reached, *T*_amb_ is the ambient temperature, *Q*_dis_ accounts for heat dissipation by the solvent and sample holder, *I* is the incident laser power, and *A*_λ_ is the sample absorbance at 785 nm (Figures 3B and S6). As a result, we obtained a moderate photothermal efficiency of 33.8% compared with other reported anisotropic plasmonic NPs.^37^ However, it is important to note that, in our system, the LSPR of the NPTs does not exactly match the laser excitation wavelength (Figure 3A), reducing absorption efficiency thereby decreasing photothermal conversion.^38^ Additionally, unlike the standardized conditions reported by Cole et al.,^37^ our experiments were conducted in a small sample volume without continuous stirring and under static, localized laser irradiation. These factors can result in pronounced thermal gradients, limited heat redistribution, and incomplete volumetric energy transfer. Thus, the observed photothermal conversion efficiency in our setup is likely underestimated compared with values attainable under fully optimized conditions.

**Figure 3:**
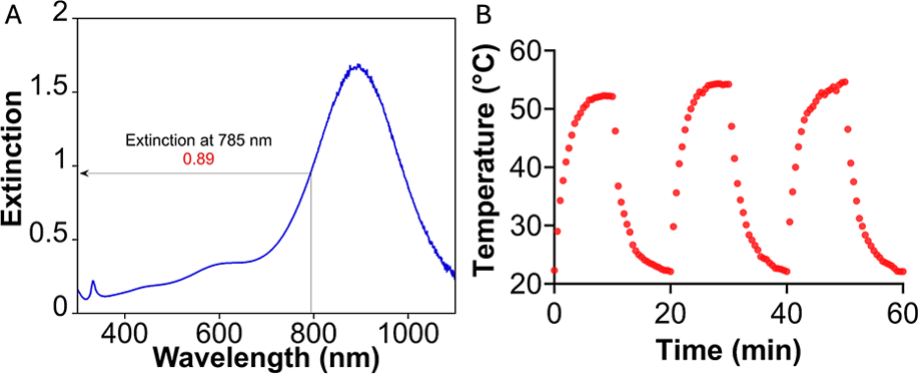
(A) Extinction spectra of the NPTs used to evaluate the photothermal conversion efficiency, which show an extinction of 0.89 at 785 nm. (B) Three consecutive heating/cooling cycles of NPTs at 2.7 W/cm^2^.

To evaluate the photothermal bactericidal efficacy of the bimetallic NPTs, a control baseline was initially verified by confirming that laser irradiation alone (20 min at 2.7 W/cm^2^) did not produce significant antibacterial effects on either of the chosen model strains, *P. aeruginosa and S. aureus* (Figure S7). Moreover, in the absence of laser exposure, the bimetallic NPTs exhibited a modest antibacterial effect against *P. aeruginosa*, noticeable only at the highest concentration tested (29.8 ppm, based on total silver content), which resulted in approximately 44% ± 3% bacterial viability. Lower concentrations showed negligible impact on bacterial survival (Figure S8A). This concentration-dependent antibacterial activity aligns with previous reports on bimetallic nanostructures composed of Ag and Au, where mechanisms such as membrane disruption, reactive oxygen species (ROS) generation, and ion release at elevated doses contribute to bacterial inactivation. The enhanced bactericidal effect under photothermal conditions underscores the synergistic role of plasmonic heating, increasing the intrinsic antibacterial properties of these NPTs.^39^

Conversely, for *S. aureus*, no significant bactericidal effect was observed at any of the tested NPT concentrations, including the highest dose (Figure S8B). This finding suggests a higher tolerance or reduced susceptibility of Gram-positive bacteria to non-photoactivated Ag–Au NPTs. The thicker peptidoglycan layer in Gram-positive bacterial cell walls likely acts as a barrier, reducing NPs penetration and limiting antibacterial efficacy. Such differential susceptibility highlights the importance of bacterial cell wall architecture in determining the effectiveness of metallic NP-based antibacterial agents.^40–42^

In contrast, laser irradiation for 20 min in the presence of NPTs resulted in complete bacterial inactivation (100% loss of viability) of both *S. aureus* and *P. aeruginosa* at 14.9 ppm Ag concentration. At a lower concentration of 7.45 ppm, the same irradiation period produced only partial bacterial lysis, with 46.3% ± 5.4% and 55% ± 4.1% bacterial viability remaining for *P. aeruginosa* and *S. aureus*, respectively (see Figure 4A,B).

**Figure 4:**
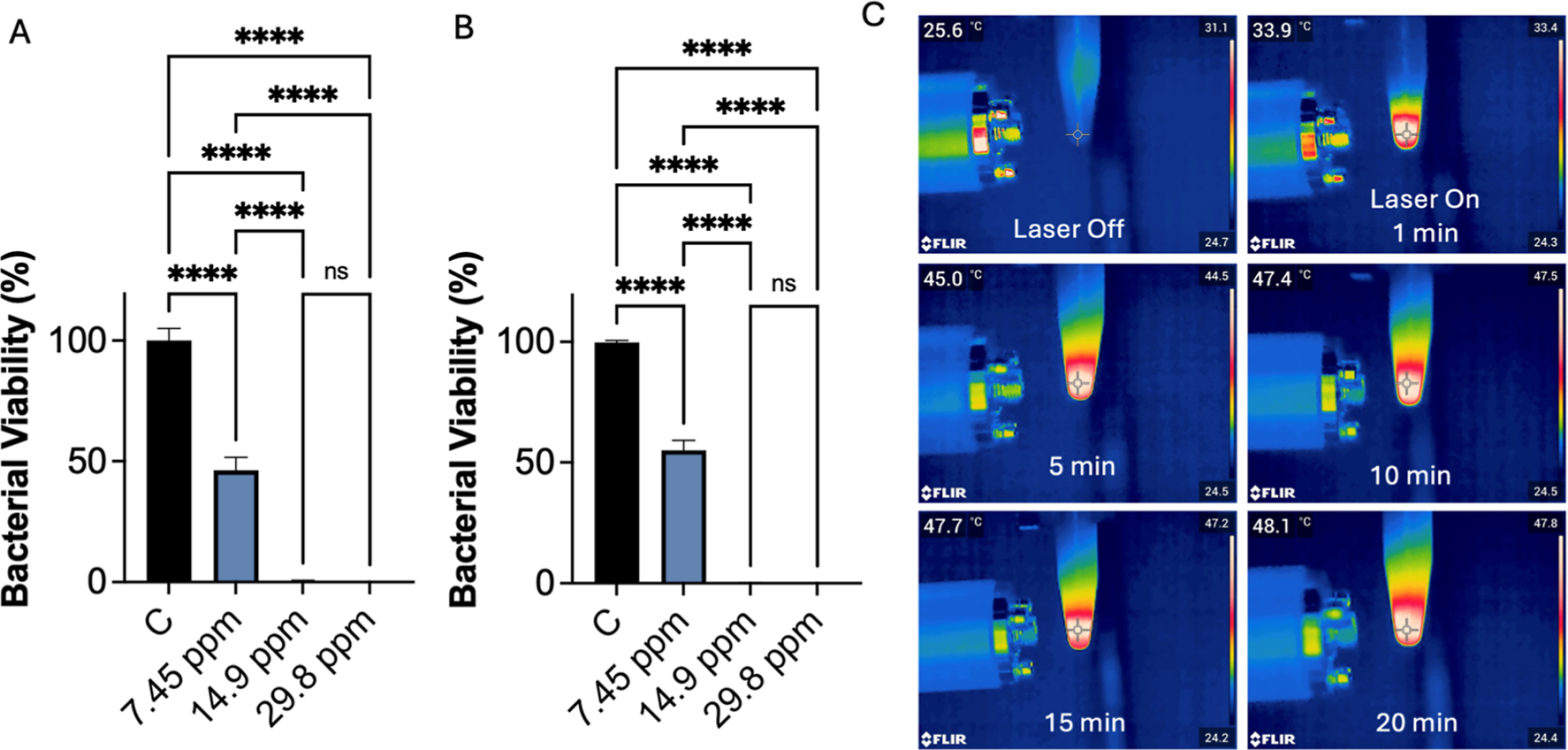
Bactericidal viability after 20 min of laser irradiation at different NPT concentrations for (A) *P. aeruginosa* and (B) *S. aureus*. Panel (C) shows an example of the temperature profile registered for *S. aureus* using NPTs at 14.9 ppm Ag concentration. Viability data corresponds to three replicates and are represented as mean ± SEM. Statistical differences were evaluated using one-way ANOVA, followed by multiple mean comparisons. ns indicates no significant difference, when evaluated at 95% confidence level; **** indicates *p* < 0.0001 in multiple comparisons.

Interestingly, the maximum temperatures reached by irradiated NPTs were consistently lower in bacterial suspensions (in PBS) than in pure aqueous solutions. For example, at an NPT concentration of 14.9 ppm Ag, the temperature of the aqueous NPT solution peaked at 56 °C, whereas the presence of bacteria resulted in a reduction of approximately 8 °C in the maximum temperature (see Figures 4C and 2A). Similar behavior was observed across all tested concentrations, with temperature drops of about 5–10 °C. This reduction in heating efficiency is likely related to the thermal properties of the bacterial suspension, which dissipates part of the photothermally generated heat,^43^ while minor differences in nanoplate dispersion between water and PBS, due to the higher ionic strength of the PBS, may also contribute to the slightly lower photothermal performance observed in bacterial suspensions.

To further explore the influence of irradiation time on photothermal antimicrobial efficacy, we varied the laser exposure times using an NPT concentration of 29.8 ppm Ag. Complete bacterial killing was achieved after just 5 min of irradiation (Figure 5A). In contrast, at 14.9 ppm concentration, reductions in *S. aureus* viability were observed in a time-dependent manner: following 5, 10, and 15 min of irradiation, bacterial viability decreased to 67.6% ± 5.5%, 40.4% ± 3.7%, and 4.3% ± 1.5%, respectively (Figure 5B,C). In the case of *P. aeruginosa* the trend is even more pronounced exhibiting increasing sensitivity under equivalent conditions; for instance, after 5 min irradiation treatments at 14.9 ppm concentration, bacterial viability dropped to just 4% ± 1.3% (Figure S9).

**Figure 5:**
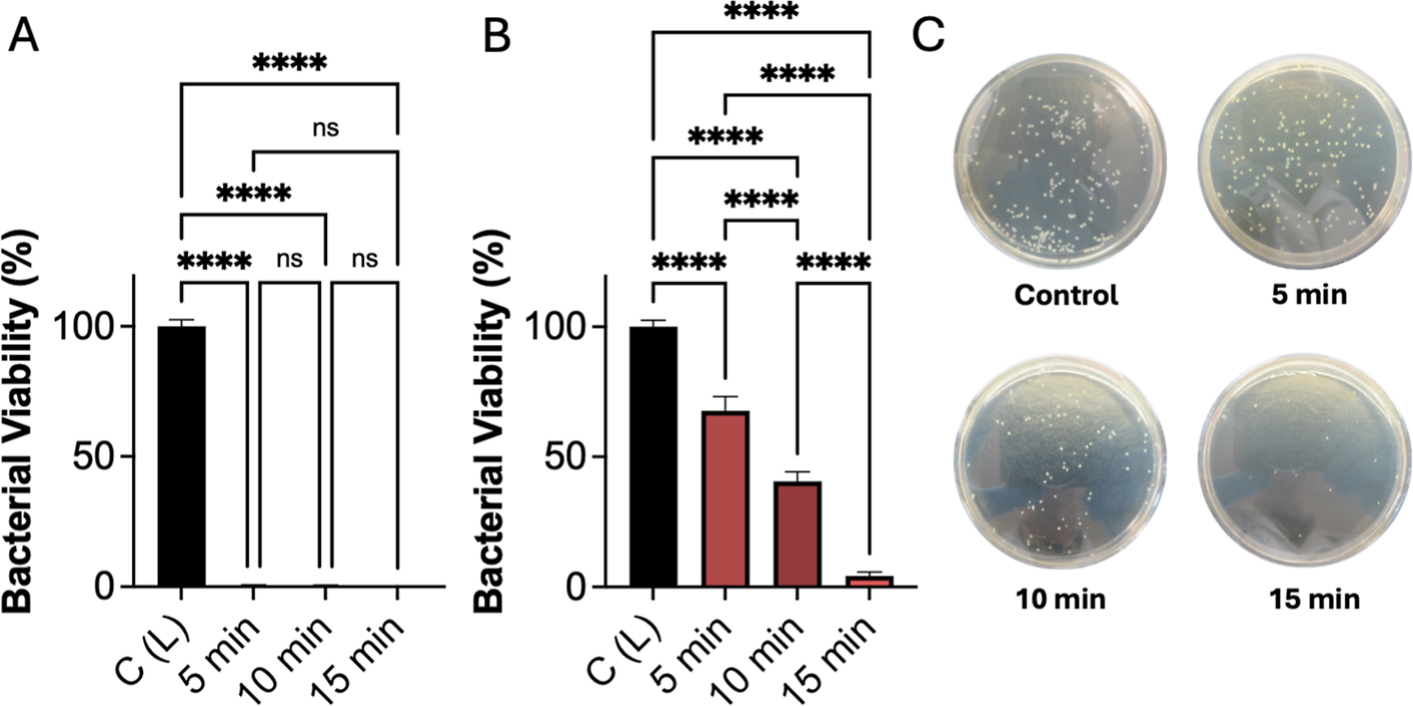
Bacterial viability of *S. aureus* after different irradiation times at NPT concentrations of (A) 29.8 and (B) 14.9 ppm Ag. (C) Representative Petri dish images corresponding to each irradiation time for *S. aureus* at 14.9 ppm of *NPT* concentration. Viability values represent the mean ± SEM of three independent replicates. Statistical differences were evaluated using one-way ANOVA, followed by multiple mean comparisons. ns indicates no significant difference at the 95% confidence level; **** indicates *p* < 0.0001 in multiple comparisons.

Considering that *P. aeruginosa* showed the highest susceptibility to PTT, this strain was selected for SEM analysis as a proof-of-concept of the underlying damage mechanism. Untreated cells exhibited the expected rod-shaped morphology with preserved surface integrity, whereas cells exposed to nanoplates and laser irradiation showed clear ultrastructural alterations, including surface damage, deformation, and focal envelope disruption (see Figure S10), in agreement with photothermal damage to the bacterial membrane, as previously reported for plasmonic nanostructure-mediated PTT against *P. aeruginosa*.^44,45^

Furthermore, we explored the photothermal antimicrobial properties of bimetallic NPTs under reduced laser power density (1.4 W/cm^2^). As expected, lowering the power density diminished the antimicrobial effect at equivalent silver concentrations. However, since the photothermal response can be effectively modulated by varying the NPT concentration (Figures 2A and 4), the antimicrobial efficacy at lower power densities can be restored by increasing NPT concentration in the medium.

To systematically investigate this effect, *S. aureus* was selected as a model organism because of its greater resistance to NPT-induced inactivation than *P. aeruginosa* (Figures 5 and S9). Initial assays evaluated the antimicrobial response of *S. aureus* to increasing NPT concentrations up to 44.7 ppm, in the absence of laser irradiation. Remarkably, *S. aureus* demonstrated substantial tolerance to NPT in solution, with 88% ± 4.5% bacterial viability maintained at the highest concentration tested without laser irradiation (Figure S11A). Upon 20 min laser irradiation, there was a complete elimination of bacteria for 44.7 ppm and near-complete bacterial eradication at 29.8 ppm (Figure S11B). The bactericidal effect demonstrated clear concentration-dependence, with bacterial viability decreasing to 64.9 ± 7.4% at an NPT concentration of 14.9 ppm (Figure S11B). Further analysis evaluated the influence of irradiation time at a fixed concentration of 44.7 ppm. Bacterial viability decreased progressively with exposure time, reaching 62.9% ± 2% after 5 min, 42.9% ± 3.7% after 10 min, and complete inactivation (0%) after 20 min (Figure S11C). Together, these findings highlight the tunable nature of photothermal antimicrobial efficacy using bimetallic NPTs, which can be precisely modulated by adjusting both irradiation parameters and/or NPT concentration.

To elucidate the mechanism underlying NPT-mediated bacterial inactivation, we investigated the possible contribution of reactive oxygen species (ROS) generated under irradiation and the potential involvement of photodynamic therapy (PDT) effects mediated by the NPTs under irradiation. Thus, we performed a photodynamic activity assay based on monitoring the UV–vis absorption of 1,3-diphenylisobenzofuran (DPBF) before and after laser exposure. Control experiments confirmed that DPBF alone remained photostable under our laser conditions, showing minimal absorbance changes (Figure S12A). When DPBF was irradiated in the presence of NPTs (with the baseline absorption of NPTs spectrally subtracted from the spectra), no measurable decrease in DPBF absorbance was observed after 10 min irradiation (Figure S12B). This result seems to indicate that the NPTs do not generate detectable singlet oxygen, and thus, exhibit negligible PDT effects under our conditions. Consequently, the antimicrobial efficacy of NPTs could be primarily attributed to photothermal heating, potentially synergized by silver ion release rather than ROS-driven photodynamic mechanisms. Our results collectively demonstrate that PEG-SH stabilization of bimetallic NPTs provides robust colloidal stability and NPT stability in biological media. However, postfunctionalization strategies offer limited scope for subsequent surface modification. In contrast, coating with silica is expected to confer equivalent or enhanced stability in biological environments while simultaneously providing a highly versatile platform for postfunctionalization. In addition to preserving the optical response, the silica shell was expected to provide a versatile platform for subsequent surface functionalization and potentially contribute to colloidal stabilization, depending on the surrounding medium.^46,47^

To evaluate the influence of a silica shell on the NPT photothermal and antimicrobial performance, we synthesized silica-coated NPTs (Ag–Au@SiO_2_) employing cetyltrimethylammonium chloride (CTAC) as a templating agent and NaOH as a catalyst, following protocols previously established for other AMP-stabilized noble metal NPs.^48–50^ After synthesis, comprehensive physicochemical characterization confirmed that the NPTs retained their LSPR after silica coating, exhibiting a characteristic redshift of approximately 40 nm (Figure 6A). Furthermore, TEM imaging revealed a continuous silica coating of NPTs, with no observable uncoated NPTs or core-free silica particles (Figure 6B,C). Additional HR-TEM images at different magnification confirmed that the Ag–Au nanoplates are surrounded by a continuous silica shell with a relatively homogeneous thickness (Figure 6D–J). Although CTAC was used as a structure-directing agent during the coating step, well-defined mesoporous channels are not clearly resolved in the high-resolution images (Figure 6I–J), suggesting a low degree of mesostructured order under the present conditions. Importantly, the side-view image shows that silica is deposited over the whole nanoplate surface, including the basal faces, and not only at the edges (Figure 6K). This interpretation is further supported by HAADF-STEM and EDS elemental mapping, where Ag remains confined to the nanoplate core (Figure 6L,M), while Si is homogeneously distributed around the particles (Figure 6N), consistent with overall silica encapsulation. The homogeneous silica shell of ca. 20 nm is consistent with optimal designs for photothermal applications.^51,52^

**Figure 6:**
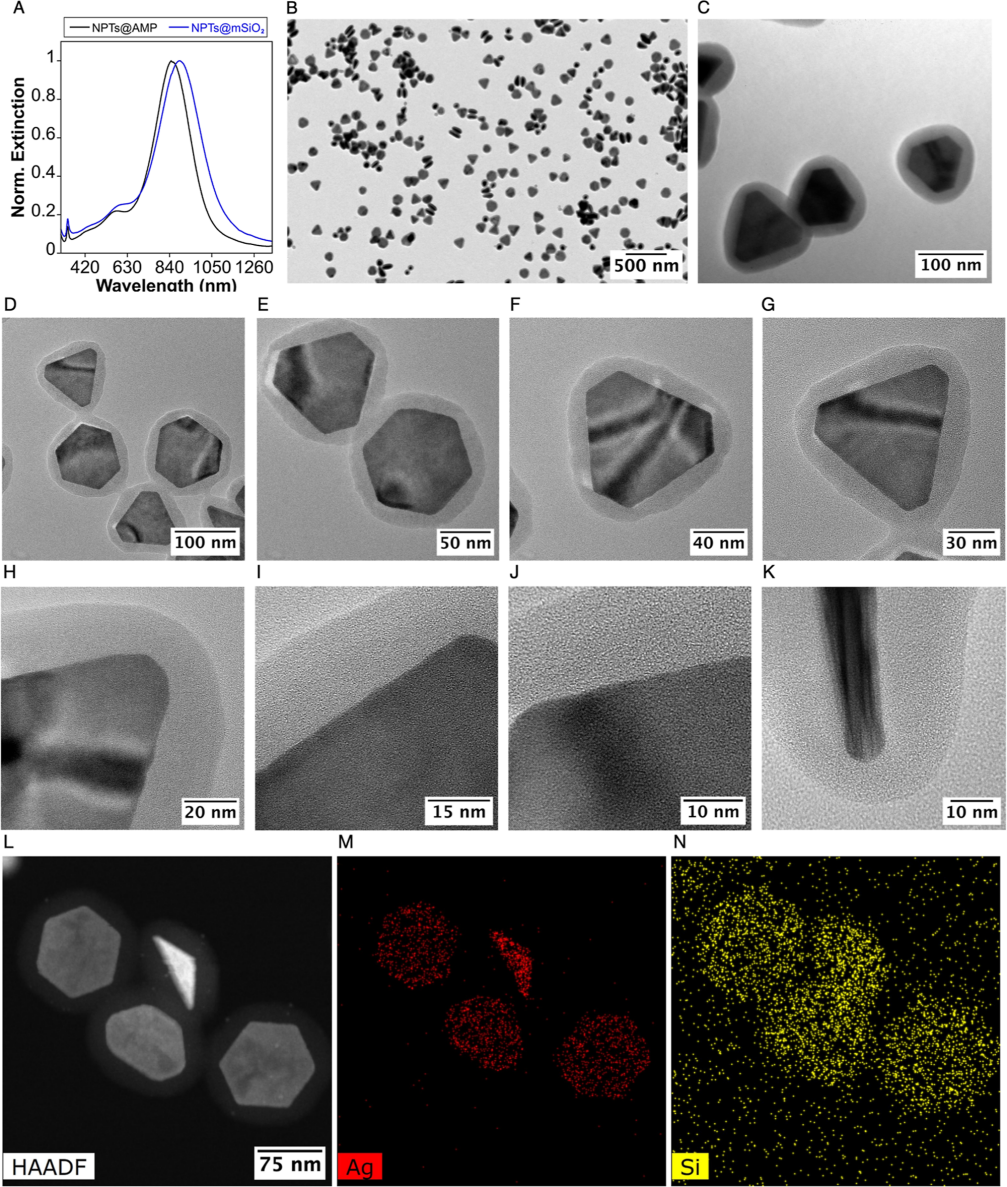
(A) Comparative normalized extinction spectra of Ag–Au@AMP and Ag–Au@SiO_2_ NPTs. (B,C) TEM images of silica-coated NPTs at different magnifications. (D–J) HR-TEM images at different magnifications showing a continuous and relatively homogeneous silica shell around the nanoplates. (K) Side-view image showing that silica covers the whole nanoplate surface, including the basal faces. (L) HAADF-STEM image of coated nanoplates. (M,N) Corresponding EDS elemental maps showing Ag confined to the nanoplate core and Si distributed throughout the surrounding shell.

We observed a decrease in the temperature generated by silica-coated NPTs compared to their counterparts stabilized solely with PEG-SH (Figure S13). To quantify the photothermal response under equivalent conditions, we systematically evaluated the conversion efficiency of silica-coated NPTs at comparable optical densities and obtained a lower value of photothermal efficiency of 27.6% (see Figure S14). This is consistent with previous reports of reduced photothermal performance of gold nanostructures following silica encapsulation.^51,53^ Such reduction is generally attributed to the limited thermal conductivity at the particle–shell interface and to increased heat dissipation through the silica matrix, which together hinder efficient heat transfer from the metal core to the surrounding medium.^53^ Recent studies further highlight the critical influence of shell composition and interfacial properties in modulating both photothermal effects and optical characteristics, underscoring the fundamental physical constraints the silica barrier imposes on nanoscale thermal transport.^54^

In addition, silica coating induces a pronounced redshift of the LSPR (≈40 nm), consistent with the higher local refractive index introduced by the silica shell.^55^ As a result, the plasmon band becomes less well matched to the 785 nm excitation wavelength, reducing optical absorption at the laser wavelength and thereby reducing the effective photothermal conversion.

In our experiments, a critical limitation was identified for silica–coated NPTs, namely their gradual coalescence in PBS over time (Figures S15 and S16). This gradual coalescence was also observed in bacterial suspensions, resulting in a decline in photothermal activity over time and variable outcomes across replicates (data not shown).

To address this challenge, the surface of Ag–Au@SiO_2_ NPTs was modified with bovine serum albumin (BSA) via covalent attachment using EDC/NHS coupling chemistry. This strategy was implemented after the silica shell of the NPTs was functionalized with (3-aminopropyl)­trimethoxysilane (APTMS) to introduce terminal amine groups. The optical response of the system remained unchanged, as shown in Figure S17A. However, we observed a pronounced inversion of the zeta potential from negative to positive values (Figure S17B). Subsequently, EDC/NHS chemistry enabled efficient BSA anchoring, as evidenced by a red shift of ca. 52 nm in LSPR band (Figure S18) and enhanced colloidal stability in PBS. This modification suppressed NPTs’ coalescence during irradiation, yielding reproducible outcomes across replicate experiments.

Consistent with previous results for uncoated NPTs stabilized by PEG-SH, silica-coated NPTs functionalized with BSA did not exhibit significant antimicrobial activity in the absence of laser irradiation, with bacterial viability remaining near 100% at either of the concentrations tested (14.9 and 29.8 ppm; Figure 7A). In contrast, comparative evaluation of photothermal antimicrobial responses revealed that BSA-functionalized Ag–Au@SiO_2_ NPTs displayed reduced activity relative to their PEG-SH-stabilized counterparts. After 20 min of irradiation, antimicrobial activity declined across all tested NPT concentrations. Specifically, as shown in Figure 7B near-complete bacterial lysis (1.3% ± 1.6% viability) required 29.8 ppm silica-BSA-coated NPTs, while a lower concentration of 14.9 ppm yielded 51.5% ± 5.8% bacterial viability. Notably, PEG-SH-stabilized NPTs at 14.9 ppm achieved much higher efficacy, reducing viability to close to zero (i.e., just 0.1 ± 0.1%, see Figure 4B). Analysis of irradiation time effects indicated that shorter laser exposures were effective only at the highest concentration tested (29.8 ppm), with bacterial viability of 40.2% ± 4.7% and 6.2% ± 3.9% observed after 5 and 10 min of irradiation, respectively (Figure 7C,D). In contrast, PEG-SH-stabilized NPTs induced nearly complete bacterial death within only 5 min of irradiation (see Figure 5A).

**Figure 7:**
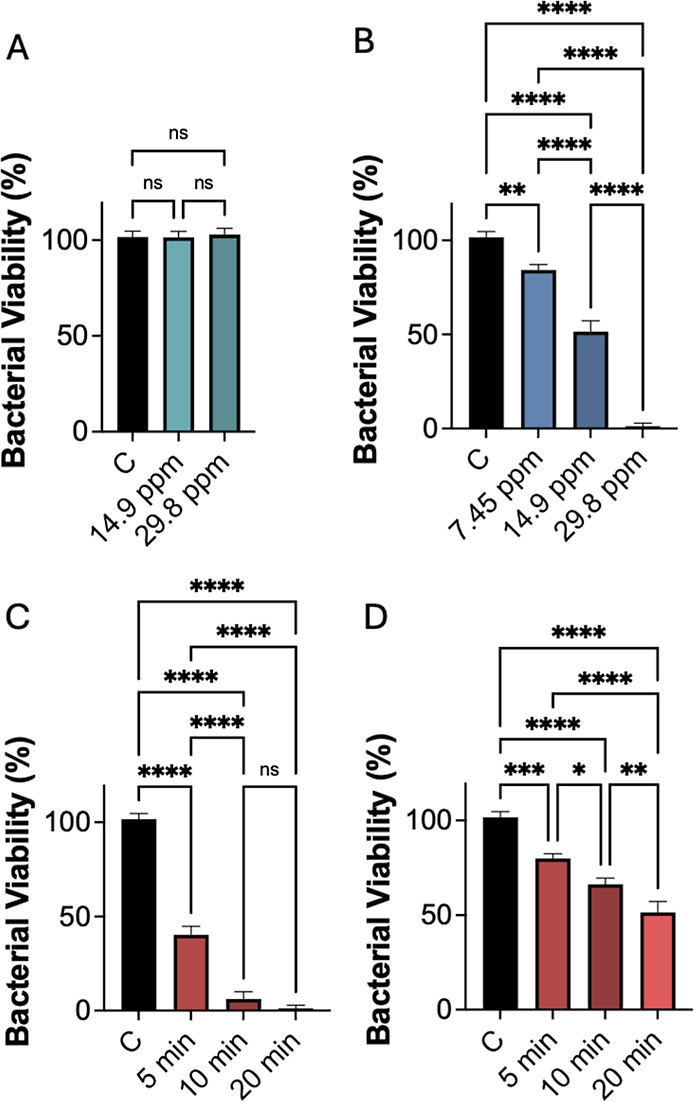
(A) Bacterial viability of *S. aureus* exposed to silica-BSA-coated NPTs at increasing concentrations without laser irradiation. (B) Bacterial viability after 20 min of laser irradiation at 2.7 W/cm^2^ at different NPT concentrations. Bacterial viability as a function irradiation time at 29.8 ppm (C) and 14.9 ppm (D) NPT concentrations. Viability data corresponds to three independent replicates and are represented as mean ± SEM. Statistical differences were evaluated using one-way ANOVA, followed by multiple mean comparisons. ns indicates no significant difference, when evaluated at 95% confidence level; * indicates *p* < 0.05 in multiple comparisons; ** indicates *p* < 0.01 in multiple comparisons; *** indicates *p* < 0.001 in multiple comparisons; **** indicates *p* < 0.0001 in multiple comparisons.

ICP measurements were performed on the supernatants collected before and after laser irradiation to assess silver release from the NPTs. In the nonirradiated condition, dissolved silver was already detected for both formulations. After irradiation, the Ag concentration in the supernatant of Ag-AuNPTs@PEG increased from 0.012 to 0.022 ppm after 10 min and to 0.039 ppm after 20 min. For Ag-AuNPTs@SiO_2_-BSA, the Ag concentration increased from 0.025 ppm before irradiation to 0.033 ppm after 10 min and to 0.042 ppm after 20 min. These results indicate an irradiation-induced silver release in both systems, with a more marked increase observed for the PEG-coated nanoplates.

The reduced photothermal antimicrobial efficacy observed for silica-BSA-coated NPTs following irradiation can be attributed to several factors: (i) diminished laser absorption due to the red-shifted LSPR at 927 nm, (ii) lower photothermal conversion efficiency relative to PEG-SH-stabilized NPTs, and (iii) potentially reduced silver release resulting from the additional silica and BSA layers. Despite these limitations, the silica-BSA-coated formulation retains substantial photothermal activity and offers the significant advantage of a highly versatile silica shell, which is well suited for applications requiring postfunctionalization. This aligns with the well-documented tunability and multifunctionality of silica platforms in biomedical systems.

An analysis of previous published studies on plasmonic NPTs under NIR irradiation indicates photothermal conversion leading to maximum temperatures typically between 55 and 80 °C.^14,18,20,21,56^ Concerning antibacterial activity, these works showed complete bacterial removal against *E. coli* and *S. aureus* using silver nanoplate concentrations between approximately 3.5 and 150 ppm under NIR laser exposure times of 5–20 min^14,18,20,21,56^

In comparison, our bimetallic NPTs demonstrate competitive performance with previously reported systems in terms of photothermal conversion efficiency (33.8%), maximum temperature achieved (up to 71 °C) and potent antibacterial activity. Additionally, our synthesis protocol enables large-batch production and facilitates the uniform deposition of a silica shell, which supports efficient protein functionalization. This combination of precise size control, reproducibility, surface engineering and multifunctional properties affords a unique and versatile platform well suited for both photothermal and chemical antimicrobial applications in biomedical contexts.

## 4 Conclusions

In summary, this work demonstrates the large-volume batch synthesis and comprehensive evaluation of bimetallic Ag–Au NPTs tailored for photothermal antibacterial therapy. PEG-SH-stabilized NPTs achieved a photothermal conversion efficiency of 33.8%, consistently reaching temperatures in water between 43 and 71 °C under NIR irradiation depending on NPT concentration and laser power density. These conditions enabled complete eradication of *P. aeruginosa* and *S. aureus* at Ag concentrations of 14.9 ppm.

Comparative studies with the silica-based platform revealed a lower photothermal conversion efficiency for the silica-coated NPTs (27.6%) and reduced antibacterial performance for the corresponding silica-BSA-coated NPTs, resulting in 51.5% ± 5.8% and 1.3% ± 1.6% viability for *S. aureus* at 14.9 and 29.8 ppm, respectively, following irradiation.

These comparative results underscore a trade-off between platform versatility and photothermal performance: silica encapsulation enables subsequent functionalization and protein conjugation, but attenuates effective heat transfer and silver release, thereby limiting bactericidal efficiency under the tested conditions. In addition, PEG-SH-stabilized NPTs showed sustained photothermal performance and minimal morphological degradation under repeated irradiation in water, whereas the silica-based platform required further surface functionalization to achieve reproducible behavior under assay-relevant conditions.

Overall, the tunability, stability, and multifunctional potential of AMP-stabilized bimetallic nanoplates provide a strong foundation for the rational design of next-generation plasmonic NPTs for antimicrobial photothermal therapy and related multifunctional antibacterial nanoplatforms.

## Supplementary Material



## Notes

Additional optical and photothermal characterization, antibacterial viability data, DPBF spectra, and physicochemical characterization of silica-coated and silica-BSA-coated nanoplates (PDF) (PDF)
