# Supplementary material for: Bimetallic Silver–Gold Nanoplates for Photothermal Antimicrobial Therapy: Integrating High Efficiency with Stable and Multifunctional Design
Source: ACS Appl Nano Mater. 2026 Apr 13;9(16):7160–72. doi: 10.1021/acsanm.6c00304 (PMC13126676; doi:10.1021/acsanm.6c00304)
Supplement: Supplementary file 1 [file an6c00304_si_001.pdf]

Supporting Information

Bimetallic Silver-Gold Nanoplates for Photothermal Antimicrobial Therapy: Integrating High Efficiency with Stable and Multifunctional Design

Javier Fernández-Lodeiro,[a,b*] Sebastian Tanco,[c,d*] Fernando Novio,[e*] Carlos Lodeiro[a,b] and Julia Lorenzo.[c,d]

[a] BIOSCOPE Research Group, LAQV-REQUIMTE, Chemistry Department, NOVA School of Science and Technology (FCT NOVA), Universidade NOVA de Lisboa, 2829-516 Caparica, Portugal.

[b] PROTEOMASS Scientific Society, 2825-466 Costa de Caparica, Portugal

[c] Institut de Biotecnologia I de Biomedicina, Departament de Bioquímica i Biologia Molecular, Universitat Autònoma de Barcelona, 08193 Cerdanyola del Vallès, Barcelona, Spain

[d] CIBER de Bioingeniería, Biomateriales y Nanomedicina, Instituto de Salud Carlos III, Campus UAB, 08913 Bellaterra, Spain.

[e] Chemistry Department, Faculty of Sciences, Universitat Autonòma de Barcelona, 08193 Cerdanyola del Vallès, Barcelona, Spain.

E-mail address: j.lodeiro@fct.unl.pt (J. Fernández-Lodeiro), Fernando.Novio@uab.cat (F. Novio), SebastianMartin.Tanco@uab.cat (S. Tanco).

KEYWORDS: Ag-Au nanoplates, plasmonic nanomaterials, silica coating, photothermal, antimicrobial.

1. Additional characterizations

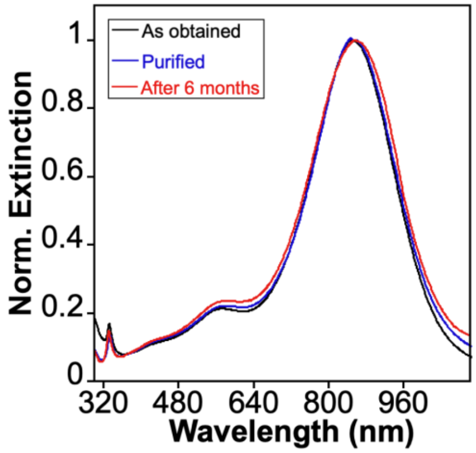

Figure S1. Comparative extinction spectra of Ag-Au@AMP NPTs as obtained, after purification, and after 6 months of storage at 4 ºC, showing largely unchanged extinction spectra with only a slight baseline increase and a small red shift (~5 nm).

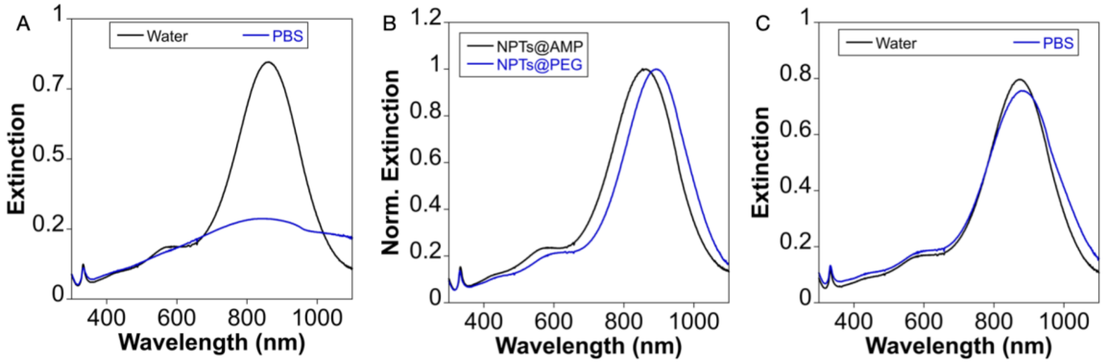

Figure S2. (A) Extinction spectra of Ag-Au NPTs stabilized with AMP/EDTA in water and PBS. (B) Normalized extinction spectra of Ag-Au NPTs stabilized with AMP or PEG-SH in water, and (C) comparative spectra of PEG-SH-stabilized NPTs in water and PBS.

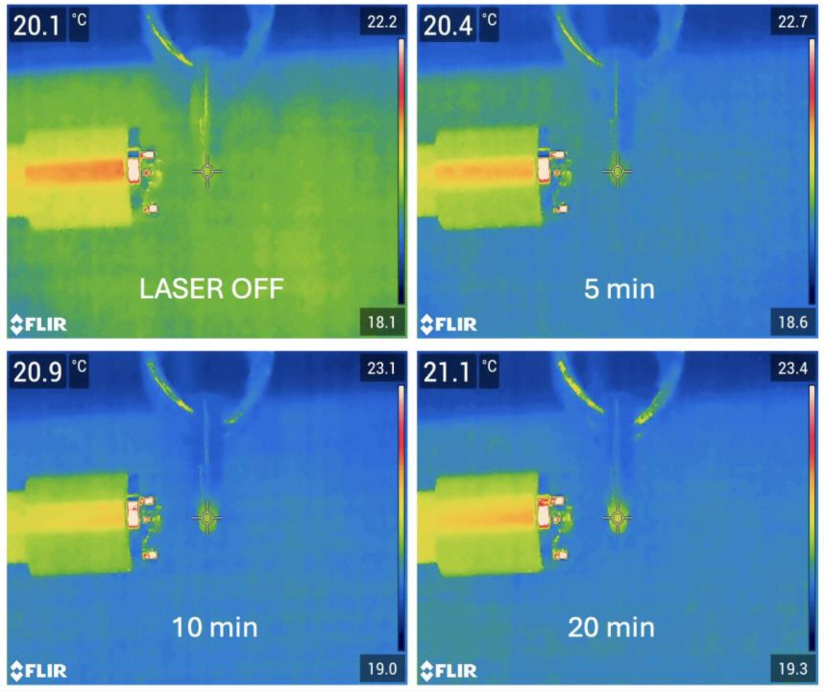

Figure S3. Thermal images of water (lacking NPTs) at different time points during irradiation at 2.7 W/cm^2.

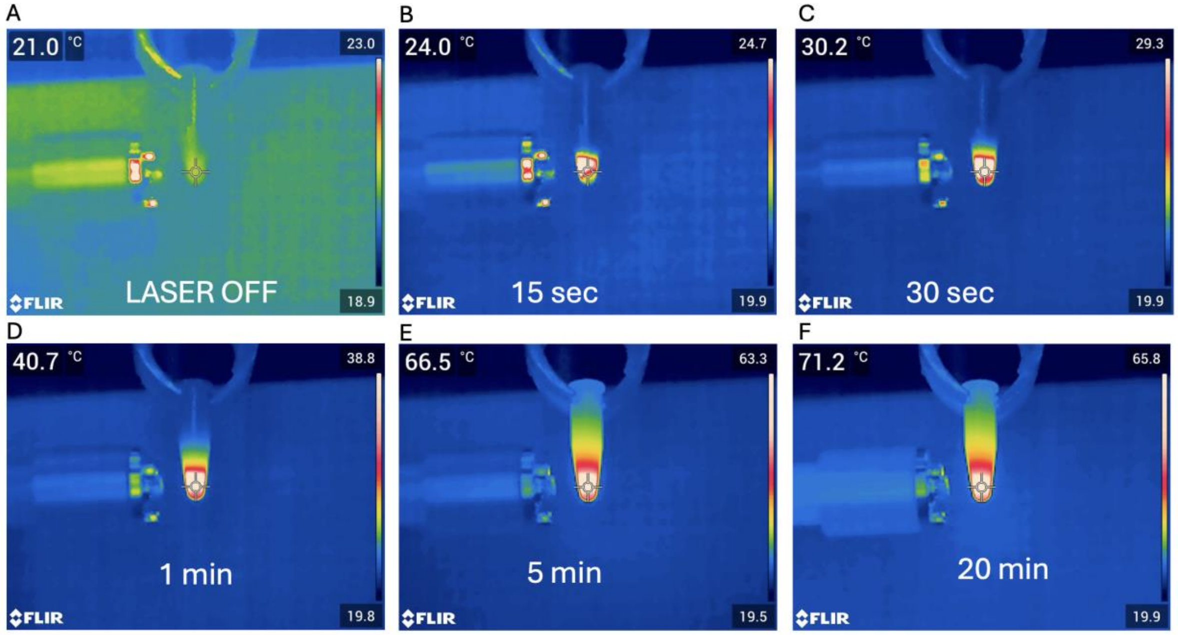

Figure S4. Thermal images of Ag-Au NPTs at 59.6 ppm Ag concentration, irradiated at different time points under 2.7 W/cm^2 laser irradiation.

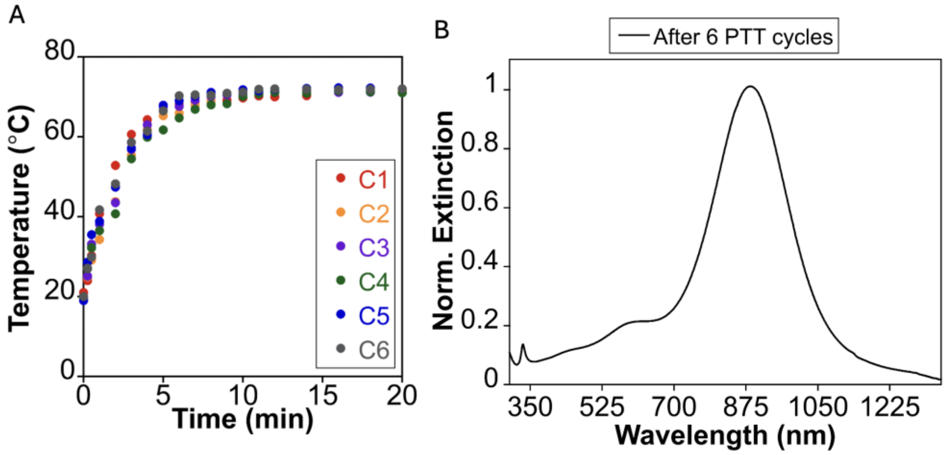

Figure S5. (A) Individual temperature profiles obtained from six independent measurements corresponding to the averaged data shown in Figure 2B. The high overlap among the six traces indicates low variability and good reproducibility of the photothermal response under the tested conditions. (B) Normalized extinction spectra of Ag-Au NPTs@PEG after 6 photothermal cycles in water showing negligible optical changes.

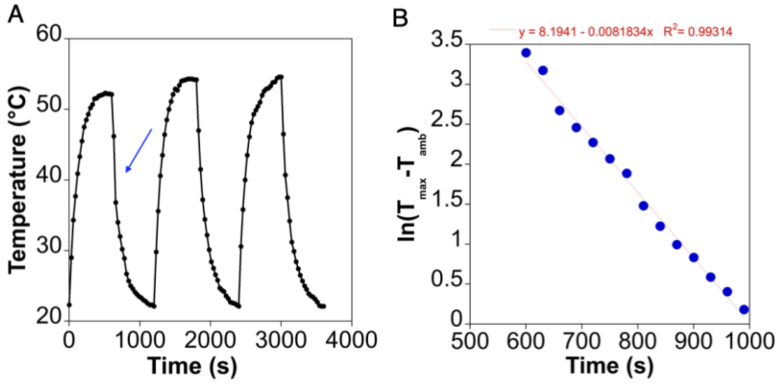

C

Parameter	Value
Excitation wavelength	785 nm
Absorbance at 785 nm	0.89
Laser power reaching the sample	0.3368 W
Cooling slope (B)	-0.0081834 s^{-1}
System time constant (τ_s)	122.2 s
Sample mass (m)	0.1 g
Heat capacity (Cp)	4.18 $Jg^{-1}K^{-1}$
Heat transfer coefficient term (hA)	0.00342 WK^{-1}
Heat dissipation (Q_{dis})	0.00342 W
Photothermal conversion efficiency	33.8%

Figure S6. (A) Heating and cooling profile of the Ag-Au NPTs dispersion recorded under 785 nm laser irradiation. The blue arrow indicates the cooling segment used for the fitting procedure. (B) Linear fit of the cooling stage according to the Roper method, used to determine the system time constant. (C) Summary of the parameters used for the photothermal conversion efficiency calculation, yielding a photothermal conversion efficiency of 33.8%.

The photothermal conversion efficiency (η) of the Ag-Au NPTs was calculated according to the Roper method from the cooling profile recorded after laser

irradiation. During the cooling stage, the dimensionless temperature-driving term θ is defined as:

$$\theta = \frac{T - T_{\mathrm{amb}}}{T_{\mathrm{max}} - T_{\mathrm{amb}}}$$

The cooling data were then linearized as:

$$-\ln(\theta) = Bt$$

where B is the slope obtained from the linear fitting of the cooling stage. From this slope, the system time constant was determined as:

$$\tau_s = -\frac{1}{B}$$

The heat transfer coefficient term was then calculated as:

$$hA = \frac{mC_p}{\tau_s}$$

Finally, the photothermal conversion efficiency was obtained from:

$$\eta = \frac{hA(T_{\mathrm{max}} - T_{\mathrm{amb}}) - Q_{\mathrm{dis}}}{I(1-10^{-A_{785}})}$$

where T is the solution temperature at a given time during cooling, T_{amb} is the ambient temperature, T_{max} is the maximum temperature reached under irradiation, B is the slope obtained from the linear fit of the cooling data, m is the sample mass, C_p is the heat capacity of the solvent, Q_{dis} is the heat dissipated by the solvent and sample holder, I is the incident laser power reaching the sample, and A_{785} is the absorbance at 785 nm.

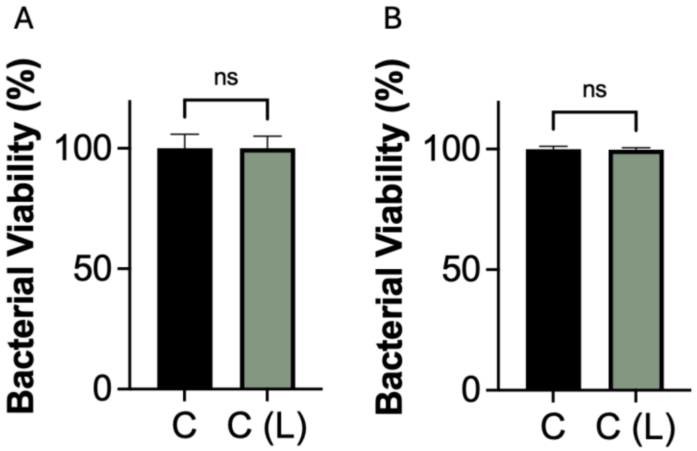

Figure S7. Effect of 20 min of laser irradiation at a power density of 2.7 W/cm² on the viability of *P. aeruginosa* (A) and *S. aureus* (B). Viability data corresponds to three replicates and are represented as mean ± SEM. Statistical differences were evaluated using a two-sided t-test. ns indicates no significant difference, when evaluated at 95% confidence level.

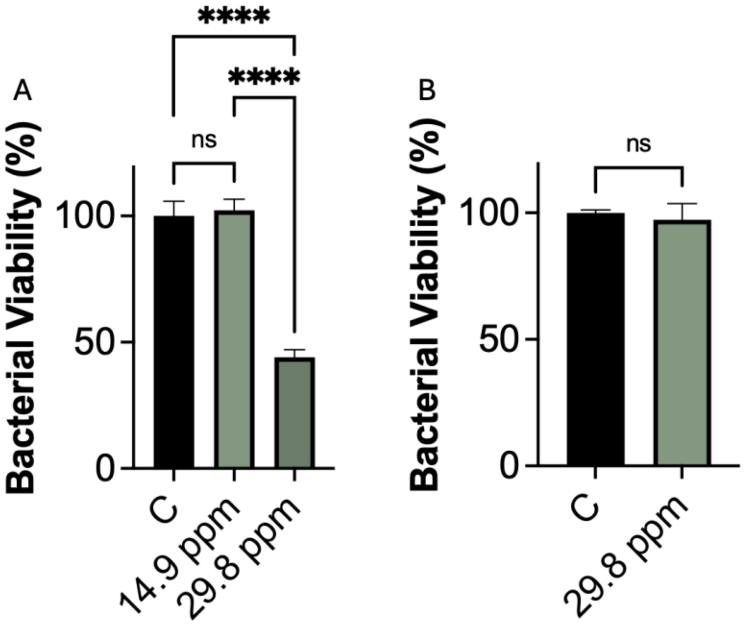

Figure S8. Effect of NPTs at different concentrations without laser input over *P. aeruginosa* (A) and *S. aureus* (B). Viability data corresponds to three replicates and are represented as mean ± SEM. Statistical differences were evaluated using one-way ANOVA, followed by multiple mean comparisons. ns indicates no significant difference, when evaluated at 95% confidence level; **** indicates $p < 0.0001$ in multiple comparisons.

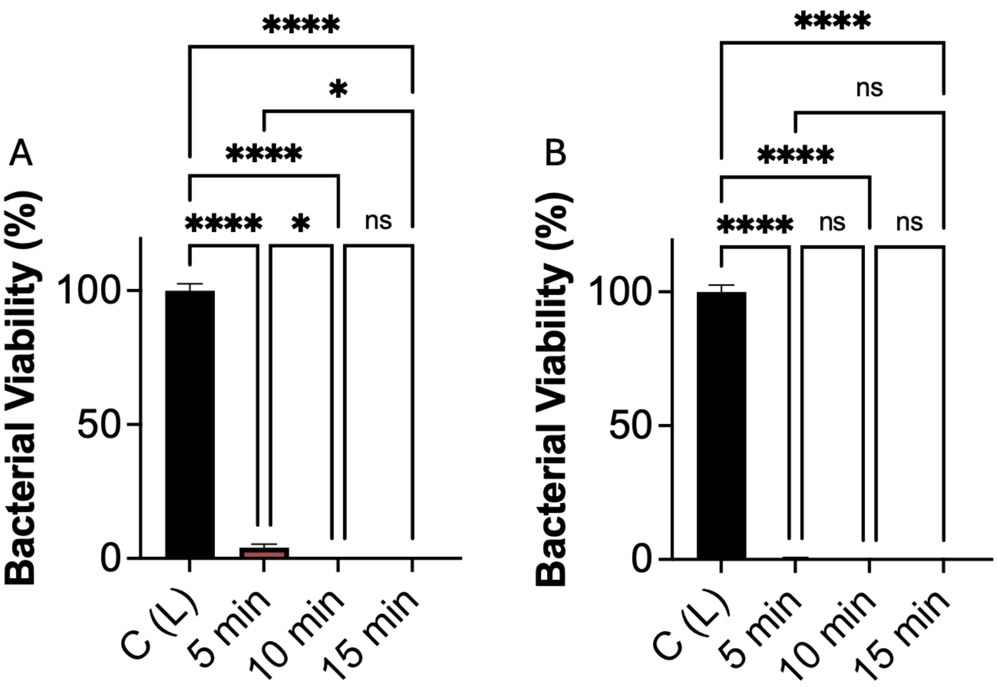

Figure S9. Bacterial viability of *P. aeruginosa* after different irradiation times at NPT concentration of 14.9 ppm (A) and 29.8 ppm (B). Viability data corresponds to three replicates and are represented as mean ± SEM. Statistical differences were evaluated using one-way ANOVA, followed by multiple mean comparisons. ns indicates no significant difference, when evaluated at 95% confidence level; * indicates $p < 0.05$ in multiple comparisons; **** indicates $p < 0.0001$ in multiple comparisons.

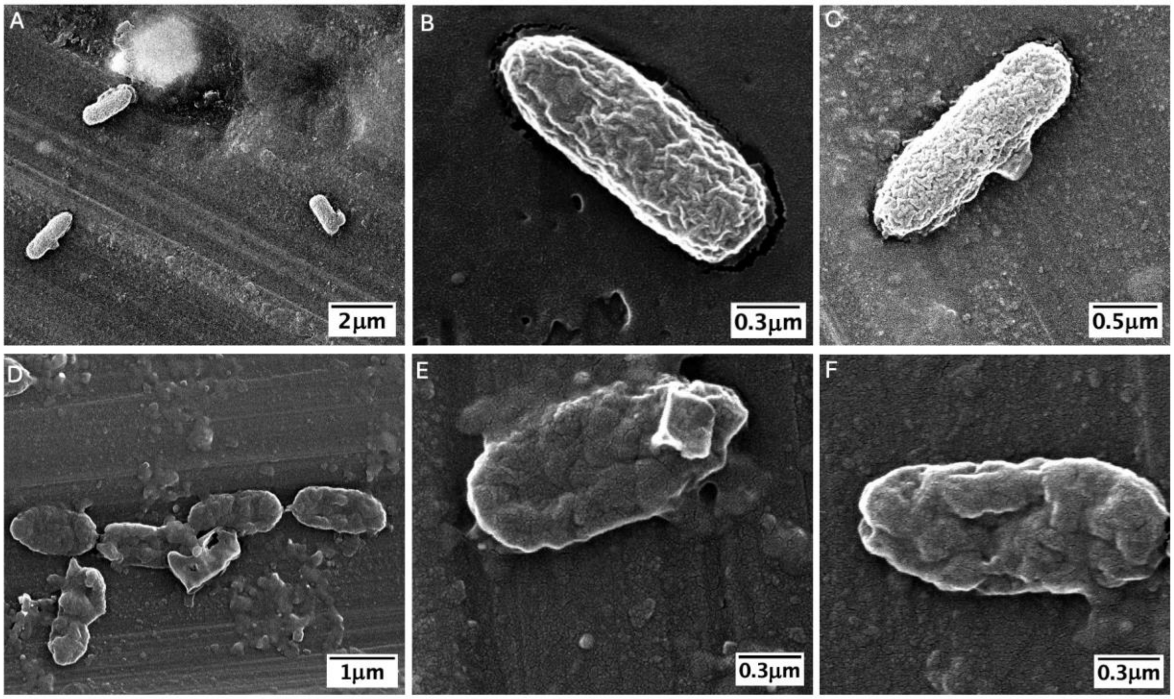

Figure S10. Representative SEM micrographs of *P. aeruginosa* control cells (A-C) and *P. aeruginosa* subjected to nanoplate-mediated photothermal treatment (D-F). While control bacteria exhibited the characteristic bacillary morphology with preserved surface integrity, treated cells displayed pronounced structural alterations, including irregular surface topology, cell deformation, and apparent envelope disruption, providing morphological evidence consistent with membrane damage induced by PTT.

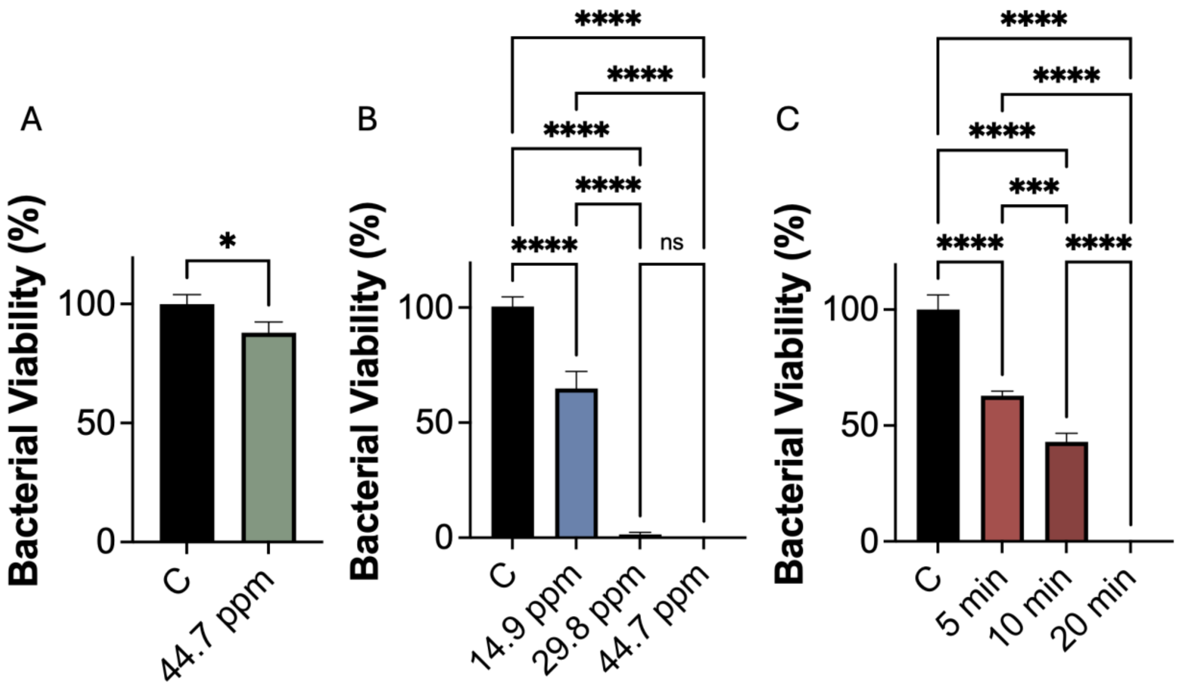

Figure S11. (A) Viability of *S. aureus* in the presence of 44.7 ppm NPT without irradiation. Viability of *S. aureus* irradiated at 1.4 W/cm^2 (B) for 20 min and at different NPTs concentrations and (C) different times of irradiation using 44.7 ppm. Viability data corresponds to three replicates and are represented as mean ± SEM. Statistical differences were evaluated using one-way ANOVA, followed by multiple mean comparisons. ns indicates no significant difference, when evaluated at 95% confidence level; * indicates $p < 0.05$ in multiple comparisons; *** indicates $p < 0.001$ in multiple comparisons; **** indicates $p < 0.0001$ in multiple comparisons.

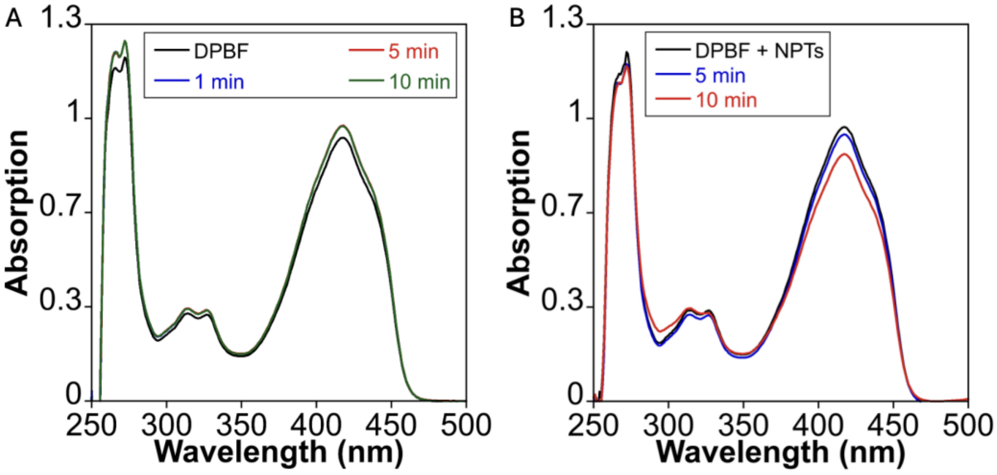

Figure S12. (A) Absorption spectra of DPBF in ACN:H_2O (60:40) before and after laser irradiation. (B) Absorption spectra of the combination of DPBF and NPTs before and after laser irradiation. (Note: A reference extinction spectrum of the NPT dispersion without DPBF, recorded under the same experimental conditions, was subtracted from the combined spectra to remove the nanoparticle baseline contribution and allow accurate analysis of DPBF spectral changes).

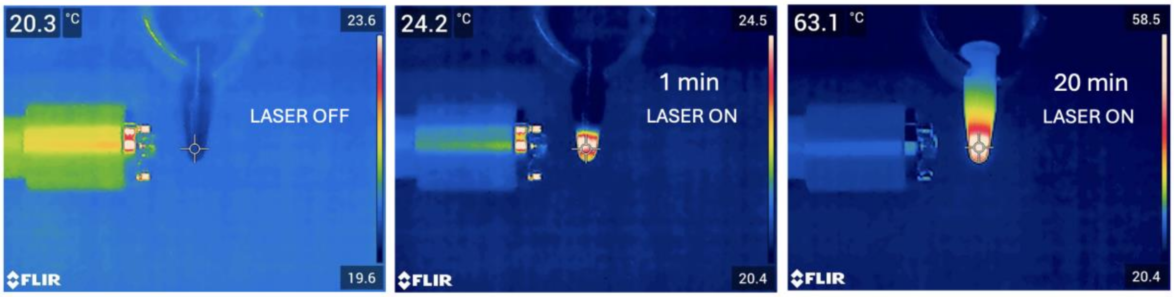

Figure S13. Representative FLIR images of the temperature profile at different times of Ag-Au@SiO_2 NPTs in water at 29.8 ppm of Ag and 2.7 W/cm^2.

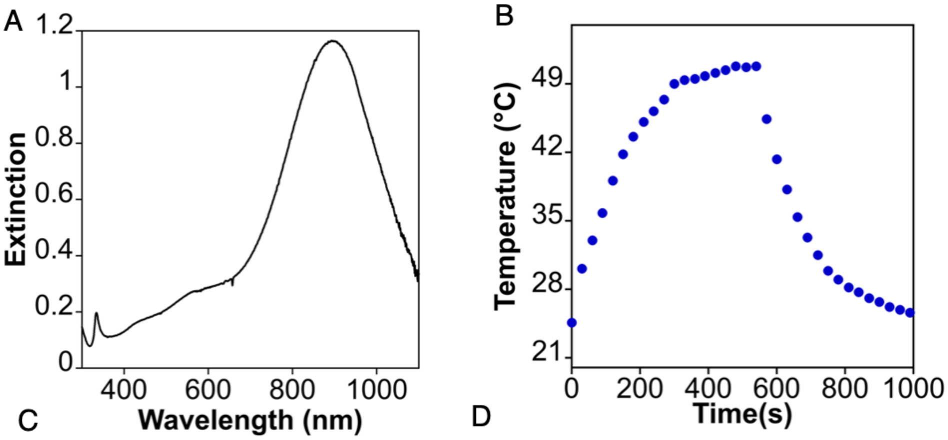

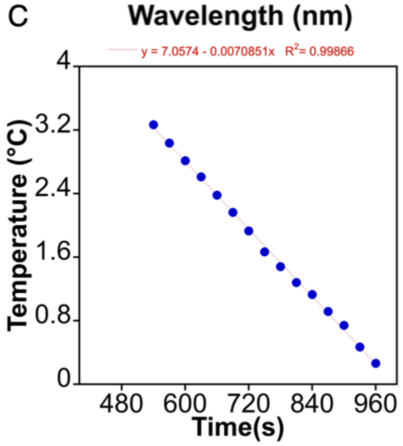

D

Parameter	Value
Excitation wavelength	785 nm
Absorbance at 785 nm	0.85
Cooling slope (B)	–0.0070851 s^{-1}
System time constant (τ_s)	141.1 s
Heat transfer coefficient term (hA)	0.00296 WK^{-1}
Photothermal conversion efficiency	27.6%

Figure S14. (A) Extinction spectrum of the silica-coated Ag-Au NPT used for the photothermal conversion efficiency measurement. (B) Heating and cooling profile of the silica-coated Ag-Au NPT dispersion recorded under 785 nm laser irradiation. (C) Linear fit of the cooling stage according to the same Roper-based method applied to the Ag-Au NPTs. (D) Summary of the sample-specific parameters used for the photothermal conversion efficiency calculation, yielding a photothermal conversion efficiency of 27.6%.

The photothermal conversion efficiency of the silica-coated Ag-Au NPTs was calculated using the same Roper-based procedure described above for the Ag-Au NPTs. Accordingly, only the sample-specific experimental data are shown here, including the extinction spectrum, the heating/cooling profile, the linear fit of the cooling stage, and the resulting calculation parameters.

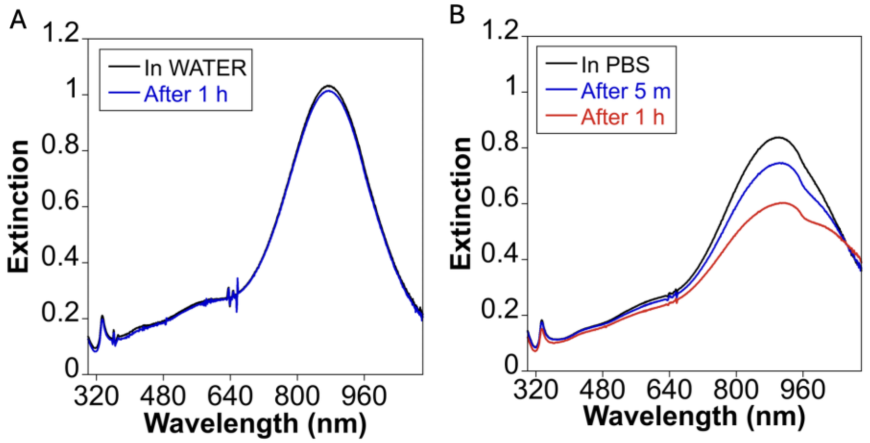

Figure S15. Comparative extinction spectra of silica-coated NPTs in water (A) and PBS (B).

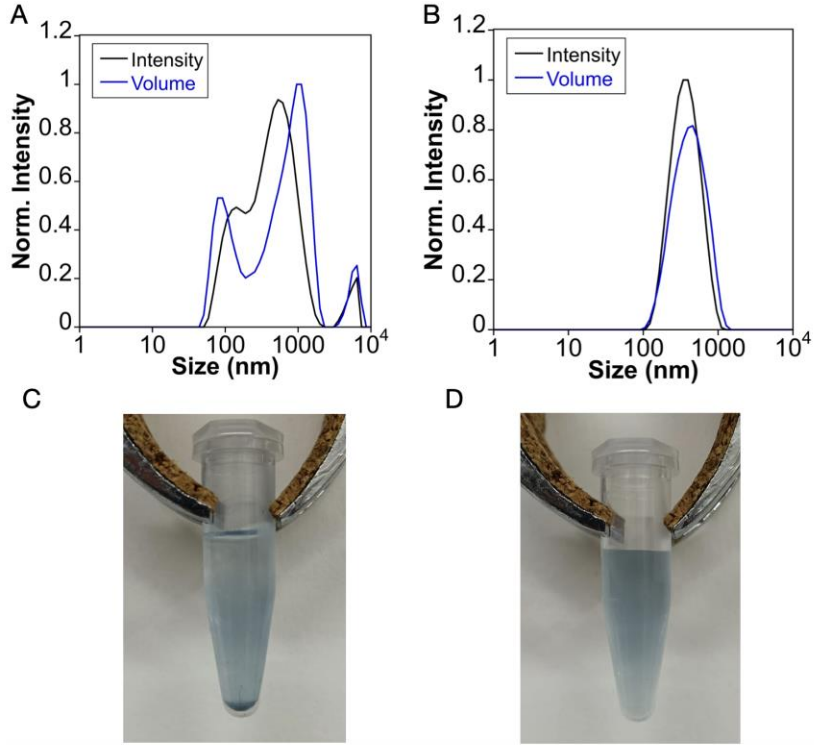

Figure S16. (A) DLS size distribution of silica-coated Ag-Au NPTs dispersed in PBS at a concentration comparable to that used in the photothermal antibacterial assays. (B) DLS size distribution of silica-BSA-coated Ag-Au NPTs dispersed in PBS at a concentration comparable to that used in the photothermal antibacterial assays. (C, D) Representative photographs of silica-coated (C) and silica-BSA-coated (D) Ag-Au NPT dispersions in PBS. These photographs were acquired using more concentrated dispersions than those employed for the DLS measurements to facilitate visual observation of sedimentation/coalescence in solution.

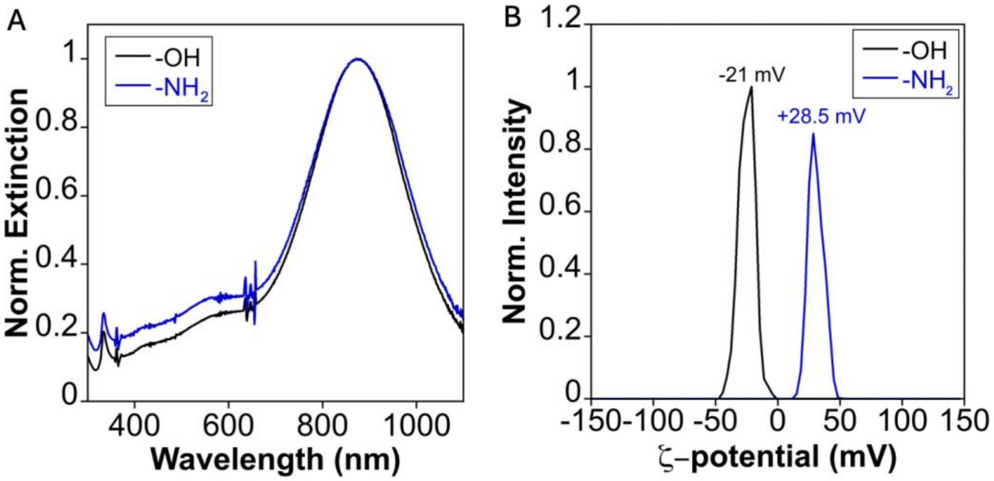

Figure S17. (A) Comparative normalized extinction spectra of NPTs coated with silica (-OH) and after APTMS condensation (-NH_2). (B) ζ potential of NPTs coated with silica (-OH) and after APTMS condensation (-NH_2).

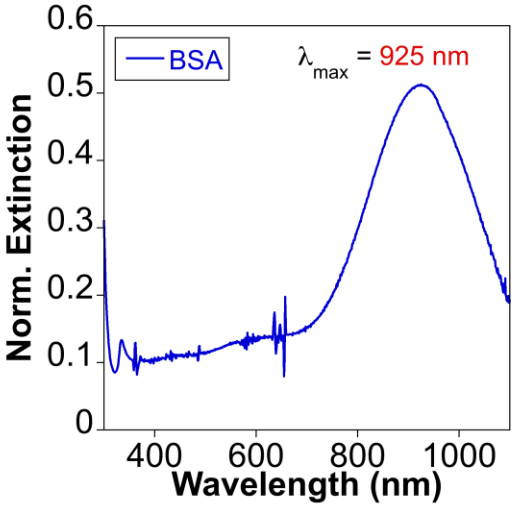

Figure S18. Extinction spectra of NPTs after BSA functionalization in PBS.
